# Conduction System vs Biventricular Pacing in Heart Failure: The PhysioSync-HF Randomized Clinical Trial

**DOI:** 10.1001/jamacardio.2026.0101

**Published:** 2026-03-11

**Authors:** André Zimerman, Alexander dal Forno, Luis E. Rohde, Caique M. Ternes, Fernanda D. Alves, Lucas P. Damiani, Martino Martinelli-Filho, Roberto Costa, Alexsandro A. Fagundes, Rodrigo M. Barbosa, Eduardo B. Gadelha, Carlos Eduardo Lima, Márcio A. Silva, Jaime A. Maldonado, Julio César de Oliveira, Fabricio Mallmann, José M. Baggio Júnior, Carlos E. Duarte, Liliane A. de Souza, Juliana S. Santos, Anderson D. Silveira, Sérgio R. R. Decker, Leandro I. Zimerman, Carisi A. Polanczyk, André d’Avila

**Affiliations:** 1 MOVE Academic Research Organization, Hospital Moinhos de Vento, Moinhos de Vento Medical School, Porto Alegre, Brazil; 2 Cardiology Division, Hospital Moinhos de Vento, Porto Alegre, Brazil; 3 Postgraduate Program in Cardiology and Cardiovascular Sciences, Federal University of Rio Grande do Sul, Porto Alegre, Brazil; 4 Research Projects Office, Hospital Moinhos de Vento, Porto Alegre, Brazil; 5 Hospital SOS Cardio, Florianópolis, Brazil; 6 Cardiology Division, Hospital de Clínicas de Porto Alegre, Porto Alegre, Brazil; 7 Brazilian Clinical Research Institute, São Paulo, Brazil; 8 Instituto Dante Pazzanese de Cardiologia, São Paulo, Brazil; 9 Department of Cardiology, Instituto do Coração, Hospital das Clínicas da Universidade de São Paulo, São Paulo, Brazil; 10 Cardiac Pacing Division, Department of Cardiovascular Surgery, Instituto do Coração, Hospital das Clínicas da Universidade de São Paulo, São Paulo, Brazil; 11 Discipline of Cardiovascular Surgery, Faculdade de Medicina FMUSP, Universidade de Sao Paulo, São Paulo, Brazil; 12 Hospital Ana Nery, Salvador, Brazil; 13 Instituto Nacional de Cardiologia, Rio de Janeiro, Brazil; 14 Instituto de Medicina Integral Professor Fernando Figueira, Recife, Brazil; 15 Hospital Universitário da Universidade Federal do Piauí, Teresina, Brazil; 16 Hospital Universitário Cassiano Antonio de Moraes, Vitória, Brazil; 17 Fundação Hospitalar do Coração Francisca Mendes, Manaus, Brazil; 18 Hospital Geral Universitário de Cuiabá, Cuiabá, Brazil; 19 Instituto de Cardiologia de Santa Catarina, São José, Brazil; 20 Instituto de Cardiologia do Distrito Federal, Brasília, Brazil; 21 Hospital Beneficência Portuguesa, São Paulo, Brazil; 22 Internal Medicine Division, Hospital Moinhos de Vento, Porto Alegre, Brazil; 23 Smith Center for Outcomes Research, Beth Israel Deaconess Medical Center, Harvard Medical School, Boston, Massachusetts; 24 Harvard-Thorndike Electrophysiology Institute, Cardiovascular Division, Department of Medicine, Beth Israel Deaconess Medical Center, Harvard Medical School, Boston, Massachusetts

## Abstract

**Importance:**

Conduction system pacing (CSP) is a promising and potentially cost-effective alternative to biventricular pacing (BiVP) in patients with heart failure with reduced ejection fraction (HFrEF) and left bundle-branch block (LBBB), but its impact on heart failure (HF) outcomes remains uncertain.

**Objective:**

To compare CSP vs BiVP on an HF-related outcome in patients with HFrEF and LBBB.

**Design, Setting, and Participants:**

PhysioSync-HF (Conduction System Pacing Versus Biventricular Resynchronization in Patients With Chronic Heart Failure) was an investigator-initiated, multicenter, noninferiority randomized clinical trial enrolling participants from November 2022 to December 2023 with 12 months of follow-up at 14 hospitals across all regions of Brazil. Adults with symptomatic HFrEF (New York Heart Association NYHA] classes II through III), left ventricular ejection fraction (LVEF) of 35% or less, and LBBB (QRS duration ≥130 milliseconds) were eligible for inclusion. Data were analyzed from May to August 2025.

**Intervention:**

Patients were randomized 1:1 to either CSP (preferentially left bundle-branch area pacing) or BiVP.

**Main Outcomes and Measures:**

The primary outcome was a hierarchical composite of death, HF hospitalizations, urgent HF visits, and change in LVEF at 12 months. The prespecified noninferiority margin for the odds ratio (OR) was 1.2.

**Results:**

A total of 173 patients (median [IQR] age, 62 years [56-68]; 86 female patients [49.7%]; 115 (66.5%) with dilated cardiomyopathy; median [IQR] LVEF, 26% [22%-31%]; median [IQR] QRS, 180 milliseconds [170-200]) were included. At 12 months, CSP failed to meet noninferiority and was inferior to BiVP for the primary end point (OR, 2.36; 95% CI, 1.37-4.06; *P* = .99 for noninferiority; *P* = .002 for between-group difference). The time-to-event composite of death, HF hospitalizations, or urgent HF visits was higher in CSP (hazard ratio, 2.35; 95% CI, 0.99-5.61). Mean (SD) LVEF increased to 35% (12%) with CSP and 39% (12%) with BiVP (mean difference, 3.8%; 95% CI, 0.3%-7.3%). Relative to baseline, both groups had comparable improvements in QRS duration, Kansas City Cardiomyopathy Questionnaire Overall Summary Score, NYHA class, and natriuretic peptide levels. Total direct medical cost related to the procedure and heart failure care was the equivalent of $7090 (95% CI, $5779-$8648) lower in patients randomized to CSP at 12 months.

**Conclusions and Relevance:**

In patients with HFrEF and LBBB, CSP was inferior to BiVP for a composite of death, HF hospitalizations, urgent HF visits, and change in LVEF at 12 months. These findings do not support the routine use of CSP as the first-line resynchronization strategy in this population.

**Trial Registration:**

ClinicalTrials.gov Identifier: NCT05572736

## Key Points

**Question:**

Is conduction system pacing (CSP) noninferior to biventricular pacing (BiVP) for patients with heart failure with reduced ejection fraction (HFrEF) and left bundle-branch block (LBBB)?

**Findings:**

In this randomized clinical trial of 173 patients across 14 sites in Brazil, CSP was inferior to BiVP for a hierarchical composite of all-cause death, heart failure hospitalizations, urgent heart failure visits, and change in left ventricular ejection fraction at 12 months.

**Meaning:**

These findings do not support the routine use of CSP as the first-line cardiac resynchronization strategy in patients with HFrEF and LBBB.

## Teaser

The PhysioSync-HF randomized clinical trial compares conduction system pacing vs biventricular pacing on heart failure–related outcomes in patients with heart failure with reduced ejection fraction and left bundle-branch block.

## Introduction

Cardiac resynchronization therapy (CRT) with biventricular pacing (BiVP) alleviates symptoms, reverses left ventricular remodeling, and lowers the risk of hospitalization and death in patients with heart failure with reduced ejection fraction (HFrEF) and intraventricular conduction delay.^1,2,3^ Yet a high proportion of patients treated with BiVP, a costly procedure, derive little benefit in heart failure outcomes, underscoring the potential for alternative strategies.^4^ Conduction system pacing (CSP) directly engages the His-Purkinje system and offers a more physiologic ventricular activation.^5^ Observational studies and small randomized clinical trials suggest that CSP can improve electrical synchrony and left ventricular function, potentially outperforming BiVP.^6,7,8,9,10,11,12,13^ Nevertheless, these studies have been limited by low patient numbers, high crossover rates, and nonadjudicated end points, leaving uncertainty about the comparative efficacy and safety of CSP over BiVP. Additionally, previous trials were largely conducted at highly selected sites in high-income countries, limiting generalizability to broader, more diverse clinical settings.

The PhysioSync-HF randomized clinical trial was designed to compare CSP with BiVP in patients with HFrEF and left bundle-branch block (LBBB). It was hypothesized that CSP would be noninferior to BiVP for a hierarchical composite of death, heart failure hospitalizations, urgent visits for heart failure, and change in left ventricular ejection fraction (LVEF) at 12 months.

## Methods

### Trial Design and Oversight

PhysioSync-HF was an investigator-initiated, multicenter randomized clinical trial comparing CSP with BiVP in patients with HFrEF and LBBB. The trial design has been described in detail previously.^14^ Patients were assigned in a 1:1 ratio to receive CSP or BiVP and followed up for 12 months. Patients and health care personnel remained blinded to group assignment, except for the implanting physicians and the immediate electrophysiology team.

PhysioSync-HF was designed and overseen by a steering committee based at Hospital Moinhos de Vento, the trial sponsor. Funding was provided by the Brazilian Ministry of Health through the Program for Institutional Development of the Unified Healthcare System. The protocol was approved by local research and ethics committees at each participating site. The trial protocol is available in Supplement 1 and the statistical analysis plan is provided in Supplement 2. An independent data and safety monitoring board, supported by an independent statistical team, reviewed unblinded data and monitored the study progress. The trial adhered to the principles of the Declaration of Helsinki and followed the Consolidated Standards of Reporting Trials (CONSORT) reporting guidelines. All authors vouch for the accuracy of the data and analyses and for the fidelity of this report to the protocol. The trial was registered at ClinicalTrials.gov (NCT05572736).

### Eligibility

Adults (aged ≥18 years) with symptomatic heart failure (New York Heart Association [NYHA] class II through III) were eligible if they had an LVEF of 35% or less, an LBBB with a QRS duration of at least 130 milliseconds, and were receiving maximum tolerated guideline-directed medical therapy, including renin-angiotensin system inhibition, a β-blocker, and a mineralocorticoid receptor antagonist. Although a strict definition of LBBB was not applied for eligibility, investigators were instructed to select patients with a QRS duration of 130 milliseconds or longer in women or 140 milliseconds or longer in men, a QS or rS pattern in leads V1 or V2, and mid-QRS notching in 2 contiguous leads (DI and aVL, V1 and V2, or V5 and V6).^15^ Patients were excluded if they had NYHA class IV symptoms, were clinically unstable, or had a plan to receive an implantable cardioverter-defibrillator with or without CRT. Complete inclusion and exclusion criteria are provided in the eMethods in Supplement 3.

Site eligibility required the presence of at least 1 board-certified physician with documented experience in both CSP and BiVP. As part of the feasibility assessment, electrocardiographic tracings from previous procedures were reviewed to confirm operator proficiency. In selected cases, members of the coordinating team conducted on-site visits to proctor initial implants and ensure adherence to protocol.

### Procedures

Intervention procedures adhered to a prespecified protocol.^14^ Devices were purchased from Biotronik through a competitive selection process, as required by the trial funder. In the CSP group, the preferred strategy was left bundle-branch area pacing; if capture criteria were not satisfied, operators sequentially advanced to His bundle pacing and, if necessary, deep septal pacing. Left bundle-branch area pacing was identified by the presence of a positive terminal wave in lead V1 along with at least 1 of the following criteria: (1) a capture pattern change from nonselective to selective or from nonselective to myocardial during threshold testing; (2) an R-wave peak time in lead V6 of 90 milliseconds or less; (3) a V6 through V1 interpeak interval of 44 milliseconds or longer; or (4) a V6 through V1 interpeak interval of 33 milliseconds or longer if the R-wave peak time in V6 was between 90 and 100 milliseconds. If electrical correction remained incomplete despite septal lead placement, adjunctive coronary sinus left ventricular pacing was permitted. The decision to implant an additional coronary sinus lead was guided by a postimplant R-wave peak time in V6 greater than 100 milliseconds or between 90 and 100 milliseconds without clear evidence of left bundle-branch capture. In the BiVP group, a right ventricular lead was combined with a left ventricular lead positioned preferentially in the basal posterolateral or lateral left ventricle. Crossover to the alternative strategy was allowed when resynchronization could not be achieved with the assigned procedure. In the CSP group, unsuccessful resynchronization was defined as failure to penetrate the septum or the complete absence of physiological correction despite attempted pacing. In the BiVP group, failure was defined as inability to cannulate the coronary sinus, lack of ventricular capture through any lead pole at 3.0 V or less at 0.4 milliseconds, or phrenic nerve stimulation in most available pacing poles.

### Outcomes

The primary end point was a hierarchical, heart failure–related composite that ranked patients according to the most severe event experienced over 12 months: (1) all-cause death; (2) hospitalization for heart failure; (3) urgent visit for heart failure, defined as an unscheduled encounter requiring intravenous diuretic therapy; and (4) change in LVEF from baseline to 12 months, grouped in 5% increments (ie, 0%-<5%, 5%-<10%). Each patient was assigned to the worst outcome within the hierarchy, with death taking precedence over all other components. Thus, every patient contributed to the primary end point: those who did not experience death or heart failure events were categorized according to their LVEF change, ranging from maximum decline to maximum improvement.

The key secondary end point was the mean total direct medical cost related to the procedure and heart failure care at 12 months. Additional secondary end points, assessed over 12 months, included changes in LVEF, left ventricular end-diastolic volume, QRS duration, natriuretic peptides, 6-minute walk distance, Kansas City Cardiomyopathy Questionnaire Overall Summary Score (KCCQ-OSS), EuroQol 5-Dimensions (EQ-5D), and NYHA class. Clinical end points comprised a time-to-first-event analysis of all-cause death, hospitalization for heart failure, or urgent visit for heart failure and a hierarchical composite of death, hospitalization for heart failure, urgent visit for heart failure, and change in the KCCQ Clinical Summary Score (KCCQ-CSS). The prespecified primary analytic cohort was the modified intention-to-treat population, defined as all randomized patients who underwent the index procedure.

An independent clinical events committee, unaware of treatment assignment, adjudicated all reported deaths, hospitalizations, and potential urgent visits for heart failure. Transthoracic echocardiograms were systematically evaluated by a central imaging core laboratory. The echocardiographic measurement protocol used the biplane Simpson method from apical 2- and 4-chamber views. The core laboratory had access only to focused echocardiographic images of the left ventricle, with no additional views available, preventing identification of pacing leads or group allocation. Twelve-lead electrocardiograms were also analyzed by an electrocardiogram core laboratory, which measured QRS duration and morphology with digital calipers under similar blinding procedures.

### Statistical Analysis

A sample size of 180 patients was calculated to provide 80% power to detect noninferiority of CSP compared with BiVP for the primary end point at 12 months. This calculation assumed, for both arms, an annual loss-to-follow-up rate of 5%, an all-cause mortality rate of 6.24%,^16^ a heart failure hospitalization rate of 5.85%,^16^ and an urgent heart failure visit rate of 0.5%.^17^ Additionally, it assumed a mean absolute difference in the 12-month LVEF of 3.0% favoring the CSP group, with a common standard deviation of 6.5.^18^

The primary end point, a composite ranging from all-cause death (worst) to the greatest improvement in LVEF at 12 months (best), was analyzed as an ordinal variable with a proportional odds mixed-effects model that included a random intercept for site and fixed effects for baseline LVEF, sex, and age. Noninferiority of CSP to BiVP would be declared if the upper bound of the 2-sided 95% confidence interval for the odds ratio (OR) was less than 1.20, corresponding to a 1-sided α of .025. If this criterion were met, a sequential test for superiority would be performed at the same α level. The key secondary end point, total direct medical costs related to the procedure and heart failure care at 12 months, was assessed by calculating the mean cost difference between treatment arms, and 95% confidence intervals were estimated with a nonparametric bootstrap with 1000 resamples.^19^ Values were converted to international dollars using the most recent World Bank purchasing power parity factor.^20^ Continuous secondary end points were evaluated with analysis of covariance, with follow-up values adjusted for respective baseline values. As exploratory analyses, the distribution of LVEF change is shown according to (1) investigator-reported resynchronization lead location and (2) the presence of a terminal R wave in lead V1 during the 12-month follow-up, a marker of conduction system capture.

## Results

### Patients

**Figure 1.**
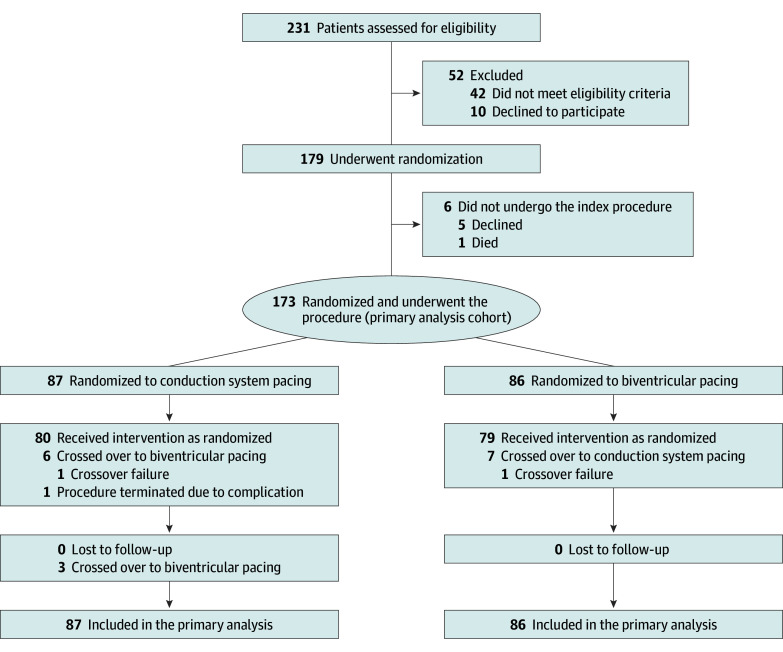
Patient Flowchart

From November 2022 through December 2023, patients were recruited at 14 sites across all regions in Brazil. A total of 179 patients were randomized, of whom 173 underwent the index procedure and were included in the primary analysis population (Figure 1). No patients were lost to follow-up, and all patients contributed to the primary end point analysis.

Patient characteristics at baseline are provided in Table 1. The median (IQR) age was 62 years (56-68), and 86 patients (49.7%) were women. Overall, for self-reported race, 34 patients (19.7%) were Black, 68 (39.3%) were multiracial, 1 (0.6%) was Native Brazilian, and 70 (40.5%) were White. The median (IQR) LVEF was 26% (22%-31%), and the median (IQR) QRS duration was 180 milliseconds (170-200). A total of 115 patients (66.5%) had dilated cardiomyopathy, and 22 (12.7%) had ischemic cardiomyopathy. Most patients were receiving guideline-directed medical therapy, with renin-angiotensin blockade prescribed in 158 patients (91.3%), β-blockers in 155 (89.6%), mineralocorticoid receptor antagonists in 144 (83.2%), and sodium-glucose cotransporter-2 inhibitors in 60 (34.7%).

**Table 1.** Baseline Characteristics

Characteristic	No. (%)	
	Conduction system pacing (n = 87)	Biventricular pacing (n = 86)
Demographics		
Age, median (IQR), y	61 (55-67)	63 (57-70)
Sex		
Female	45 (51.7)	41 (48)
Male	42 (48.3)	45 (52)
Race^a^		
Black	16 (18.4)	18 (20.9)
Multiracial	34 (39.1)	34 (39.5)
Native Brazilian	0	1 (1.2)
White	37 (42.5)	33 (38.4)
Body mass index, median (IQR)^b^	27.6 (25.1-30.4)	26.5 (23.4-31.8)
Comorbidities		
Hypertension	58 (66.7)	53 (61.6)
Diabetes	35 (40.2)	30 (34.9)
Atrial fibrillation	7 (8.0)	5 (5.8)
Heart failure etiology^c^		
Dilated cardiomyopathy	53 (60.9)	62 (72.1)
Ischemic	13 (14.9)	9 (10.5)
Hypertensive	13 (14.9)	7 (8.1)
Chagasic	4 (4.6)	2 (2.3)
Valvular	5 (5.7)	2 (2.3)
Alcoholic	2 (2.3)	4 (4.7)
Other or unspecified	1 (1.1)	5 (5.8)
Symptoms and functional status		
NYHA functional class		
I	1 (1.1)	0
II	47 (54.0)	45 (52.3)
III	39 (44.8)	41 (47.7)
6-min Walk distance, median (IQR), m^d^	350 (234-412)	320 (242-413)
EQ-5D score, median (IQR)	0.60 (0.45-0.69)	0.53 (0.46-0.66)
KCCQ-OSS, median (IQR)	39 (25-52)	35 (22-51)
Baseline test values, median (IQR)		
QRS duration, ms	180 (170-200)	180 (170-200)
Typical LBBB (Strauss criteria), No. (%)	84 (96.6)	81 (94.2)
Left ventricular ejection fraction, %	26.0 (22.8-31.3)	26.9 (21.6-31.5)
Left ventricular end-diastolic volume, mL	196 (155-265)	184 (154-251)
BNP, pg/mL^e^	315 (114-616)	222 (77-476)
NT-proBNP, pg/mL^e^	1302 (682-2522)	1056 (329-2797)
Medical therapy		
ACEi, ARB, or ARNI	81 (93.1)	77 (89.5)
ARNI	35 (40.2)	38 (44.2)
β-Blocker	79 (90.8)	76 (88.4)
Mineralocorticoid receptor antagonist	68 (78.2)	76 (88.4)
SGLT2 inhibitor	25 (28.7)	35 (40.7)

Abbreviations: ACEi, angiotensin-converting enzyme inhibitor; ARB, angiotensin receptor blocker; ARNI, angiotensin receptor–neprilysin inhibitor; BNP, brain natriuretic peptide; EQ-5D, EuroQol 5-Dimensions; KCCQ-OSS, Kansas City Cardiomyopathy Questionnaire Overall Summary Score; LBBB, left bundle-branch block; NT-proBNP, N-terminal prohormone of brain natriuretic peptide; NYHA, New York Heart Association; SGLT2, sodium-glucose cotransporter-2.

SI conversion factor: To convert BNP from pg/mL to ng/L, multiply by 1.

^a^ Self-reported.

^b^ Calculated as weight in kilograms divided by height in meters squared.

^c^ Heart failure etiologies are not mutually exclusive.

^d^ Three patients did not undergo the 6-minute walk test due to physical limitations.

^e^ Sites were allowed to measure either BNP (n = 81) or NT-proBNP (n = 91).

### Procedure

Procedural characteristics are summarized in eTable 1 in Supplement 3. The index procedure was performed a median (IQR) period of 2 days (1-6) after randomization, and 99 cases (57.2%) were performed by operators who had at least 40 prior CSP cases. The overall median (IQR) procedure duration was 120 minutes (80-164), and fluoroscopy time was 20 minutes (12-33), with no differences between study arms. Crossover during the index procedure was attempted in 6 of 87 patients assigned to CSP (6.9%) and 7 of 86 patients assigned to BiVP (8.1%). Three patients did not receive a study device. Among the 80 patients who received CSP per protocol, lead position was classified by the operator as left bundle-branch area in 55 patients (68.8%), deep septal in 16 (20.0%), left bundle area pacing with optimized resynchronization in 7 (8.8%), and His bundle in 2 (2.5%).

The median (IQR) postprocedure QRS duration was 120 milliseconds (103-133) in patients randomized to CSP (a median [IQR] reduction of 60 milliseconds [45-80] from baseline) and 126 milliseconds (118-138) in the BiVP group (a median [IQR] reduction of 55 milliseconds [36-71]). In patients randomized to CSP, electrophysiologic markers of acute resynchronization and conduction system capture included a postprocedure median (IQR) R-wave peak time in V6 of 83 milliseconds (70-105) and a V6 through V1 interpeak interval of 42 milliseconds (25-63). In the BiVP arm, the median (IQR) postprocedure QLV interval was 121 milliseconds (98-150). Acute pacing thresholds were comparable between groups (CSP: median, 0.8 V; IQR, 0.5-1.0; BiVP: 0.8 V; IQR, 0.6-1.4).

### Outcomes

**Figure 2.**
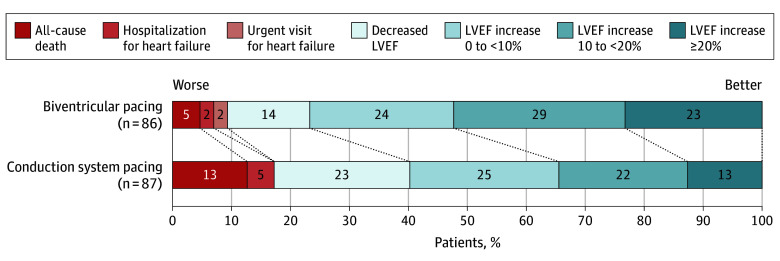
Component Bar Graph Showing Primary End Point Results^a^ Shown are the distribution of outcomes at 12 months. Patients were categorized according to the worst outcome they experienced during follow-up, with death taking precedence over all other components. Patients who did not experience clinical events were categorized based on their left ventricular ejection fraction (LVEF) change at 12 months. LVEF was analyzed in 5% increments, as described in the Methods; for the graphical presentation, adjacent categories were merged to improve visual interpretability. Cell values represent the rounded percentage of patients in each category. An odds ratio (OR) value >1.0 favors the biventricular pacing group. ^a^OR, 2.36; 95% CI, 1.37-4.06.

**Figure 3.**
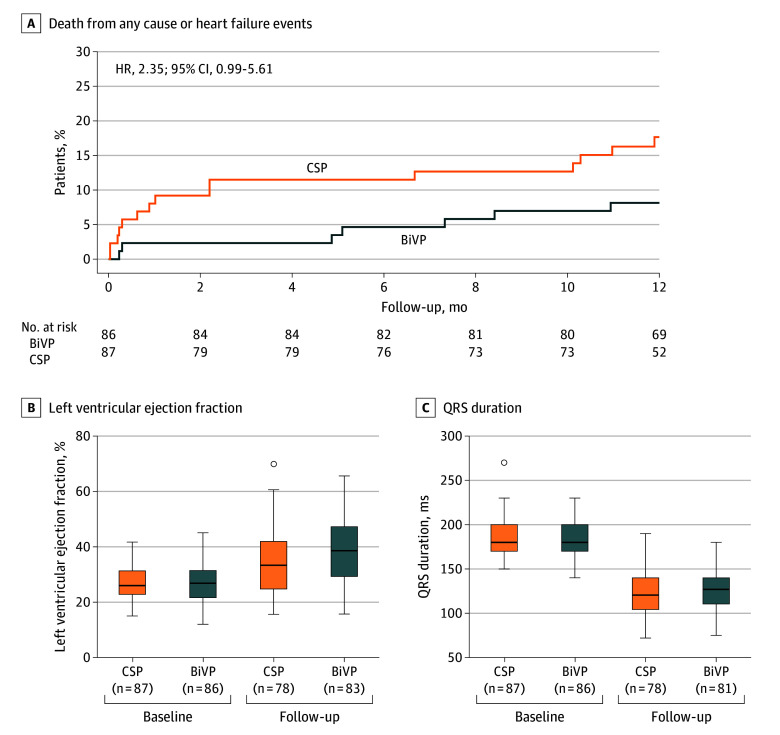
Kaplan-Meier Survival Plot and Box Plots of Secondary End Points A, Cumulative incidence of all-cause death or heart failure events, which include hospitalizations and urgent visits for heart failure. B, Left ventricular ejection fraction. C, QRS duration. Box plots show the median (horizontal line), interquartile range (box), and minimum and maximum values excluding outliers (whiskers); individual outliers are plotted as points. Follow-up was up to 12 months after the index procedure; in cases of unavailable data at 12 months, the corresponding 6-month value was imputed per the Statistical Analysis Plan. BiVP indicates biventricular pacing; CSP, conduction system pacing; HR, hazard ratio.

At 12 months, CSP did not meet the prespecified noninferiority criterion and was inferior to BiVP for the hierarchical primary end point (OR, 2.36; 95% CI, 1.37-4.06; *P* = .99 for noninferiority; *P* = .002 for between-group difference) (Figure 2, Table 2). Findings were consistent in the per-protocol analysis (eTable 2 in Supplement 3). The secondary end point of all-cause death, hospitalization for heart failure, or urgent heart failure visit occurred in 15 patients in the CSP group (17.2%) and 8 patients in the BiVP group (9.3%) (hazard ratio [HR], 2.35; 95% CI, 0.99-5.61) (Figure 3). During the trial, 11 patients in the CSP group (12.6%) died compared with 4 in the BiVP group (4.7%) (HR, 3.36; 95% CI, 1.05-10.71). Individual causes of death are detailed in eTable 3 in Supplement 3.

**Table 2.** Primary and Secondary End Points^a^

End point	No./total No. (%)		Effect measure	Effect (95% CI)
	Conduction system pacing (n = 87)	Biventricular pacing (n = 86)		
Primary end point				
All-cause death, hospitalization for heart failure, urgent visit for heart failure, and change in left ventricular ejection fraction	NA	NA	OR	2.36 (1.37 to 4.06)
All-cause death	11/87 (12.6)	4/86 (4.7)	HR	3.36 (1.05 to 10.71)
Hospitalization for heart failure	6/87 (6.9)	4/86 (4.7)	HR	2.20 (0.60 to 8.02)
Urgent visit for heart failure	1/87 (1.1)	2/86 (2.3)	HR	0.59 (0.05 to 6.68)
Change in left ventricular ejection fraction, mean (SD), %	8 (11)	12 (11)	Mean difference	−3.8 (−7.3 to −0.3)
Secondary end points				
Total direct medical cost, US $^b^	15 666	22 756	Mean difference	−7090 (−8648 to −5779)
All-cause death, hospitalization for heart failure, or urgent visit for heart failure	15/87 (17.2)	8/86 (9.3)	HR	2.35 (0.99 to 5.61)
Left ventricular ejection fraction, mean (SD), %	35 (12)	39 (12)	Mean difference	−3.8 (−7.3 to −0.3)
Left ventricular end-diastolic volume, mean (SD), mL	175 (77)	171 (90)	Mean difference	−3.7 (−25.6 to 18.2)
QRS duration, mean (SD), ms	122 (25)	126 (21)	Mean difference	−4.5 (−11.7 to 2.6)
Relative difference in BNP from baseline, median (IQR), %^c^	−55 (−75 to −8)	−48 (−86 to 4)	Mean difference	−0.5 (−92.9 to 92.0)
Relative difference in NT-proBNP from baseline, median (IQR), %^c^	−48 (−82 to 28)	−52 (−86 to −16)	Mean difference	12.3 (−48.1 to 72.8)
6-min Walk distance, mean (SD), m	390 (103)	358 (100)	Mean difference	33.1 (3.0 to 63.2)
KCCQ-OSS, mean (SD)	75 (25)	77 (21)	Mean difference	−1.5 (−8.5 to 5.6)
EQ-5D score, mean (SD)	0.70 (0.32)	0.73 (0.23)	Mean difference	−0.03 (−0.12 to 0.05)
All-cause death, hospitalization for heart failure, urgent visit for heart failure, and change in KCCQ Clinical Summary Score	NA	NA	OR	1.33 (0.78 to 2.26)
NYHA class				
I	36/73 (49.3)	38/82 (46.3)	OR	1.02 (0.53 to 1.98)
II	31/73 (42.5)	39/82 (47.6)		
III	6/73 (8.2)	5/82 (6.1)		
IV	0/73	0/82		

Abbreviations: BNP, brain natriuretic peptide; EQ-5D, EuroQol 5-Dimensions; HR, hazard ratio; KCCQ-OSS, Kansas City Cardiomyopathy Questionnaire Overall Summary Score; NA, not applicable; NT-proBNP, N-terminal prohormone of brain natriuretic peptide; NYHA, New York Heart Association; OR, odds ratio.

^a^ Follow-up was up to 12 months after the index procedure; for continuous variables, in cases of unavailable data at 12 months, the corresponding 6-month value was imputed per the Statistical Analysis Plan. For the primary end point, patients were categorized according to the worst outcome they experienced during follow-up, and change in left ventricular ejection fraction from baseline was analyzed in 5% increments.

^b^ Converted from Brazilian reals using a purchasing power parity factor of 2.44.

^c^ Sites were allowed to measure either BNP (n = 81) or NT-proBNP (n = 91).

Compared with baseline, improvements were observed across all secondary end points in both randomized groups (Table 2). However, the increase in LVEF was attenuated with CSP compared with BiVP, with a between-group difference of 3.8% (95% CI, 0.3%-7.3%). No between-group differences were observed in improvements in left ventricular end-diastolic volume, QRS duration, brain natriuretic peptide (BNP) levels, N-terminal prohormone of BNP (NT-proBNP) levels, KCCQ-OSS, EQ-5D scores, NYHA functional class at 12 months, or in a composite of clinical events and change in KCCQ-CSS from baseline. The increase in 6-minute walk distance was more pronounced in the CSP group. Moreover, the total direct medical cost related to the procedure and heart failure care was estimated to be the equivalent of $7090 (95% CI, $5779-$8648) lower in patients randomized to CSP compared with BiVP at 12 months of follow-up.

Results of the primary end point across prespecified patient subgroups are shown in eTable 4 in Supplement 3. Significant interactions were observed for age and heart failure etiology, indicating a more substantial benefit of BiVP in patients aged 62 years or older and with nonischemic disease. Changes in LVEF according to lead location and the presence of a terminal R wave in lead V1 during follow-up are shown in eFigures 1 and 2 in Supplement 3, respectively.

### Safety

A total of 10 patients in the CSP group (11.5%) and 7 patients in the BiVP group (8.1%) experienced at least 1 procedure-related complication, most commonly lead dislodgement or fracture (eTable 1 in Supplement 3). Three procedure-related deaths occurred, all in patients randomized to the CSP group, although 1 case occurred after crossover to BiVP (eTable 5 in Supplement 3). These events included a sudden pulseless electrical activity arrest after a seemingly uncomplicated implant, a suspected right ventricular perforation with subsequent multiorgan failure, and tamponade during attempted coronary sinus cannulation resulting in cardiogenic shock. Late crossovers occurred in 3 patients initially assigned to CSP and no patients assigned to BiVP, such that attempted crossovers ultimately occurred in 9 (10.3%) and 7 (8.1%) patients, respectively.

## Discussion

In patients with symptomatic HFrEF and LBBB, CSP was inferior to BiVP on a hierarchical composite outcome of all-cause death, heart failure events, and change in LVEF at 12 months. The odds of a worse heart failure–related outcome were approximately 2-fold higher with CSP compared with BiVP, driven by a higher incidence of clinical events as well as a lesser improvement in LVEF.

For over 2 decades, BiVP has been the cornerstone of cardiac resynchronization in patients with heart failure and electrical dyssynchrony, based on the clinical benefits demonstrated in landmark trials.^1,2^ Recently, CSP has emerged as a promising alternative, supported by favorable results from small randomized studies.^6,7,8,9,10,11,12,13^ Considering these preliminary data, international guidelines are cautiously endorsing CSP as an alternative to BiVP, particularly in patients with symptomatic HFrEF and LBBB,^21,22^ and the uptake of CSP in clinical practice has expanded rapidly.^23^

The unanticipated results of the PhysioSync-HF trial and their apparent discordance with previous studies could be attributed to several nonexclusive mechanisms. First, the suboptimal outcomes of the CSP arm could be attributed to inadequate implant technique, a recurrent criticism in explaining its poor performance. In our study, which included 14 sites with heterogeneous background CSP experience, periprocedural complications were high and more frequent in the CSP arm, and clinical event curves diverged shortly after implant. Although the excess risk for the primary end point remained statistically significant after removing procedure-related deaths, one cannot exclude that a steeper learning curve may have negatively impacted the CSP arm. Nearly 20% of patients randomized to CSP had deep septal pacing, which may be less effective than left bundle-branch area pacing and could have contributed to an attenuated response. Notably, however, CSP led to a decrease in QRS that was comparable or superior to that in prior studies, indicating effective resynchronization, and both groups exhibited clinically meaningful improvements in secondary end points. Second, patients enrolled in PhysioSync-HF were more likely to be female, have nonischemic LBBB, and advanced left ventricular remodeling than in prior studies, and this patient population is known to display a robust response to BiVP. In the present analysis, BiVP conferred a significantly greater benefit in patients with nonischemic disease than in those with ischemic disease, suggesting that patients with diffuse myocardial disease may require the broader electrical engagement conferred by BiVP. Third, all echocardiographic, electrocardiographic, and clinical end points were evaluated by independent, blinded committees, minimizing the likelihood of ascertainment bias that may have favored CSP in previous studies. Nevertheless, the absolute number of patients and clinical events remained modest compared with cardiovascular outcomes trials. Finally, the exclusive use of stylet-driven leads, which have been associated with higher rates of lead dislodgement and complications than lumenless systems in some studies, may have disadvantaged the CSP group.^24,25^

Low-income countries exhibit higher rates of major cardiovascular events and procedure-related complications than high-income countries, reflecting a combination of later-stage disease at presentation, limited access to perioperative and critical care, and variability in clinician expertise and health care infrastructure.^26,27^ Consistent with these observations, we noted 3 procedure-related deaths—a high rate that lacked a single unifying cause. Results from PhysioSync-HF suggest that the effectiveness of technically demanding therapies, such as CRT, may not translate across diverse settings, underscoring the need for standardized implementation and dedicated procedural trials in low- and middle-income countries.^28^ With regard to CRT, our findings carry immediate policy implications: although CSP has been promoted as a potentially cost-saving alternative to BiVP, particularly in low- and middle-income countries, our data suggest that CSP should not yet become the standard approach for CRT in these environments.

### Limitations

The PhysioSync-HF trial has limitations. First, while the clinical and geographical diversity of patients enhanced generalizability, it also introduced challenges in standardizing care delivery across sites. Second, although all implanting physicians had prior experience with CSP or underwent formal proctoring, the cumulative experience with CSP was less than with BiVP, and a learning curve effect among operators and health care systems cannot be excluded. The MELOS registry suggested that 100 to 200 cases may be required to achieve proficiency in left bundle-branch area pacing^23^; this threshold exceeded the experience of most operators in PhysioSync-HF, underscoring how results may not directly reflect those of highly experienced centers. Third, PhysioSync-HF was a moderately sized trial with relatively few clinical outcomes; thus, the possibility that our findings reflect random variation must be acknowledged, and further large-scale studies are warranted.^29^ Fourth, patients with a plan to undergo implantable cardioverter-defibrillator therapy were excluded due to the prohibitive costs associated with device implantation, and results may not be generalizable to this subgroup or patients with ischemic cardiomyopathy.

## Conclusions

In the PhysioSync-HF randomized clinical trial among patients with HFrEF and LBBB, CSP failed to meet noninferiority and was inferior to BiVP for a composite of all-cause death, heart failure hospitalizations, urgent visits for heart failure, and LVEF change at 12 months.

## Notes

Supplement 1. Trial Protocol

Supplement 2. Statistical Analysis Plan

Supplement 3. eAppendix 1. Trial Leadership and Committees eAppendix 2. Site Investigators eMethods. Additional Details on Methods eTable 1. Procedural Characteristics and Complications eTable 2. Sensitivity Analyses for the Primary End Point eTable 3. Individual Causes of Death eTable 4. Subgroup Analysis for the Primary End Point eTable 5. Narrative Summaries of Deaths Associated With Procedural Complications eFigure 1. Change in Left Ventricular Ejection Fraction by Resynchronization Lead Location eFigure 2. Change in Left Ventricular Ejection Fraction at 12 Months by Presence of Terminal Positive Wave in Lead V1

Supplement 4. Data Sharing Statement
