# Supplementary material for: Conduction System vs Biventricular Pacing in Heart Failure: The PhysioSync-HF Randomized Clinical Trial
Source: JAMA Cardiol. 2026 Mar 11;11(4):360–8. doi: 10.1001/jamacardio.2026.0101 (PMC12980360; doi:10.1001/jamacardio.2026.0101)
Supplement: Supplement 1. — Trial Protocol [file jamacardiol-e260101-s001.pdf]

HOSPITAL MOINHOS DE VENTO

PHYSIOSYNC-HF PROTOCOL

Conduction System Pacing Versus Biventricular Resynchronization in Patients with Chronic Heart Failure
PhysioSync-HF

PORTO ALEGRE, NOVEMBER 21, 2024

This document is an English translation of the original Protocol, which was written and applied in Portuguese.

HOSPITAIS PROADI-SUS
PROADI SUS
SUS
MINISTÉRIO DA SAÚDE
GOVERNO FEDERAL BRASIL UNIÃO E RECONSTRUÇÃO

PROTOCOL TITLE

Conduction System Pacing Versus Biventricular Resynchronization in Patients with Chronic Heart Failure

TRIAL SPONSOR

Hospital Moinhos de Vento, Porto Alegre, Brazil.

Funded by a grant from the Brazilian Ministry of Health through the Program for Institutional Development of the Unified Healthcare System (PROADI-SUS).

STUDY COORDINATING SITE

Hospital Moinhos de Vento, Porto Alegre, Brazil.

Funded by a grant from the Brazilian Ministry of Health through the Program for Institutional Development of the Unified Healthcare System (PROADI-SUS).

CLINICAL TRIALS ID

NCT05572736

ABBREVIATIONS

6MWT – Six-Minute Walk Test
ACS – Acute Coronary Syndrome
AE – Adverse Events
BNP – B-type Natriuretic Peptide
CEC – Clinical Events Committee
CI – Confidence Interval
CPET – Cardiopulmonary Exercise Test
CRF – Case Report Form
CRT – Cardiac Resynchronization Therapy
GCP – Good Clinical Practice
HF – Heart Failure
HFrEF – Heart Failure with Reduced Ejection Fraction
HMV – Hospital Moinhos de Vento
HUB – Specialized Study Site
ICD – Implantable Cardioverter-Defibrillator
IRB – Institutional Review Board
LBBB – Left Bundle Branch Block
LVAT – Left Ventricular Activation Time
LVEF – Left Ventricular Ejection Fraction
NYHA – New York Heart Association Functional Class
OR – Odds Ratio
PROADI-SUS – Institutional Development Support Program of the Unified Health System
SUS – Brazilian Unified Public Health System
TDABC – Time-Driven Activity-Based Costing

ABSTRACT

This study aims to evaluate whether conduction system pacing via the His-Purkinje network is clinically non-inferior and economically superior to conventional biventricular pacing in cardiac resynchronization therapy (CRT) for patients with congestive heart failure and complete left bundle branch block (LBBB). A prospective, randomized, actively controlled clinical trial, blinded to participants and endpoint assessors will be conducted. The study is designed as a non-inferiority trial for the primary clinical endpoint. Participants will be randomized in a 1:1 ratio using a centralized, web-based system with allocation concealment. Data will be analyzed on an intention-to-treat basis. Eligible participants include adults (≥18 years) with symptomatic heart failure (NYHA class II–III) despite optimized medical therapy, left ventricular ejection fraction (LVEF) ≤35%, and complete LBBB with QRS duration ≥130 ms. Exclusion criteria include NYHA class IV heart failure, planned ICD implantation, life expectancy <1 year, pregnancy, current participation in another clinical trial, or refusal to provide informed consent.

Following randomization, participants will be assigned to either the intervention group (conduction system pacing) or the control group (standard biventricular pacing with a cardiac resynchronization device). Follow-up visits will occur in person at 30 days, 6 months, and 12 months. The primary endpoint is a hierarchical composite clinical outcome assessed on an ordinal scale including all-cause mortality, heart failure hospitalization, urgent visit for heart failure, and change in LVEF at 12 months. A total of 180 patients will be required to assess non-inferiority with 80% statistical power. This study is part of the Institutional Development Support Program of the Brazilian Unified Health System (PROADI-SUS) and aligns with the strategic priorities of the Technical Division of Science and Technology, Ministry of Health (Brazilian Federal Official Gazette – NUP: 25000.123471/2021-59).

Figure 1. PhysioSync-HF Flowchart

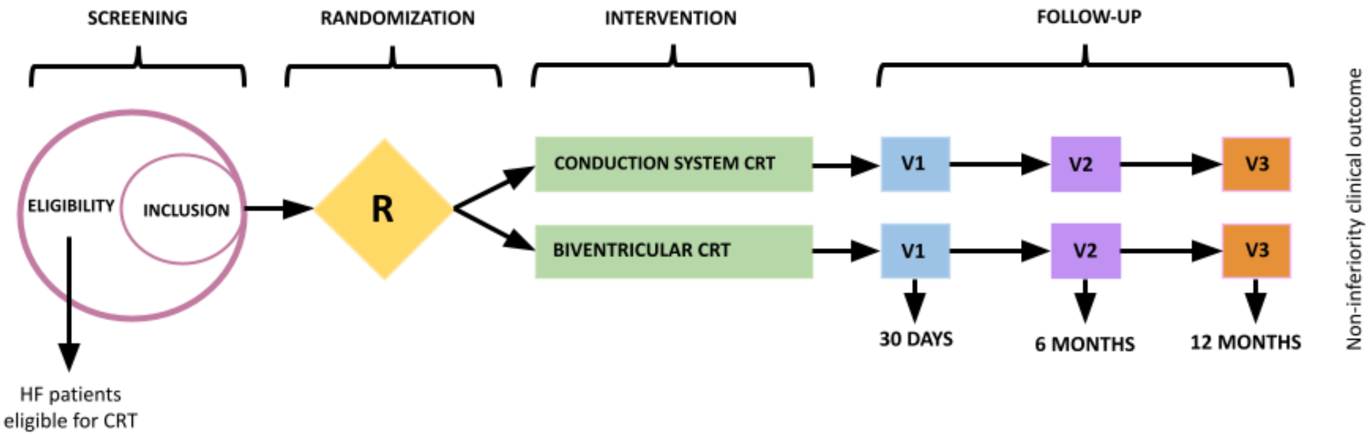

Abbreviations: CRT – cardiac resynchronization therapy; HF – heart failure; V1 – first in-person outpatient visit; V2 – second in-person outpatient visit; V3 –third in-person outpatient visit.

SUMMARY OF KEY CHANGES 7
BACKGROUND 8
OBJECTIVES 10
2.1 Primary Objective 10
2.2 Conceptual Hypothesis 10
2.3 Secondary Objectives 10
STUDY DESIGN 11
3.1 Design 11
3.2 Eligibility 11
3.2.1 Eligibility Criteria for Participating Hospitals 11
3.2.2 Eligibility Criteria for Participating Patients 11
3.2.2.1 Inclusion Criteria 11
3.2.2.2 Exclusion Criteria 12
3.3 Outcomes and Events 12
3.3.1 Primary Outcome 12
3.3.2 Secondary Outcomes 13
3.3.3 Exploratory Outcomes 13
3.3.4 Safety Outcomes 14
3.3.5 Adjudicated Outcomes 15
3.3.6 Adverse Events 15
3.4 Randomization and Allocation Concealment 17
3.5 Blinding 17
3.6 Study Procedures 17
3.6.1 His-Purkinje Conduction System Pacing Protocol 19
3.6.2 Follow-up 21
3.6.3 Visit Description 21
3.6.4 Management of Patients in Case of Incorrect Inclusion (or Randomization) 22
3.6.5 Data Collection for Economic Analysis 23
3.7 Concomitant Therapies 24
3.8 Risks and Benefits to Patients 24
3.9 Risks and Benefits to the Brazilian Unified Health System (SUS) 25
3.10 Follow-up 25

3.11 Loss to Follow-up 25
3.12 Sample Size Calculation 26
3.13 Statistical Analysis Plan 27
3.14 Data Collection System 27
3.15 Recruitment 28

ETHICAL CONSIDERATIONS AND GOOD CLINICAL PRACTICE 29
4.1 Local Study Approval 29
4.2 Informed Consent 29
4.3 Central Study Approval 29

STUDY COORDINATION 30
5.1 Coordinating Center 30
5.2 Steering Committee 30
5.3 Executive Committee 30
5.4 Publication Committee 30
5.5 Adjudication Process 30
5.6 Data Quality Management 31
5.7 Independent Data and Safety Monitoring Board 32
5.8 Sponsor Responsibilities 32
5.9 Responsibilities of Investigators and Subinvestigators 33
5.10 Monitoring 33
5.11 Publication of Results 33
5.12 Protocol Amendments 33

REFERENCES 34

SUMMARY OF KEY CHANGES

Topic	Description of Change	Timing
**Primary Endpoint Definition**	The protocol initially defined co-primary endpoints: an ordinal clinical composite and a cost-effectiveness analysis. This was revised to designate the clinical composite as the primary endpoint, with the economic analysis reclassified as a key secondary endpoint.	July 2022, prior to enrollment of the first participant.
**Primary Endpoint Structure**	Originally, LVEF change was categorized as: <0%, 0–7.9%, 7.9–14.4%, and >14.4%. In the revised version, the ordinal scale was restructured into 5% increments (i.e., –10 to –5%, –5 to 0%, 0 to +5%, +5 to +10%, etc.).	July 2022, prior to enrollment of the first participant.
**Design**	Clarified that the trial employed patient blinding.	July 2022, prior to enrollment of the first participant.
**Statistical analysis**	The initial calculation assumed a co-primary endpoint, with a one-sided alpha of 4% and a noninferiority margin defined as an odds ratio <1.1. This was revised to reflect the updated design (clinical endpoint as primary), adopting a noninferiority margin of odds ratio <1.2.	July 2022, prior to enrollment of the first participant.
**Sample size**	Sample size was revised from 304 to 180 patients based on updated assumptions and evidence published mid-enrollment.	June 2023, after 87 patients had been randomized and none had completed 12-month follow-up.

1. BACKGROUND

Heart failure (HF) patients with limited response to Guideline-Directed Medical Therapy (GDMT) remain at high risk of morbidity, mortality, and adverse left ventricular remodeling (1,2). Randomized controlled trials (RCTs) have demonstrated a net clinical benefit of cardiac resynchronization therapy (CRT) in these patients, particularly in those with reduced ejection fraction and a wide QRS complex and left bundle branch block (LBBB) (3–5). However, 30–40% of the population receiving CRT fail to show clinical improvement with biventricular pacing and continue to impose a high-cost burden on the Brazilian public health system (SUS) (6–8).

As an alternative to conventional resynchronization—typically performed by implanting one lead in the right ventricular endocardium and a second lead in a coronary sinus branch—direct stimulation of the His-Purkinje system can be used, targeting either the His bundle or the left bundle branch (9–11). In His bundle pacing, the right ventricular lead is fixed near the apex of the Koch's triangle, allowing for selective or non-selective capture of the His-Purkinje system. By engaging the heart's native conduction system, this technique restores physiological ventricular activation and may prevent undesirable effects of conventional biventricular pacing, such as ventricular remodeling (12).

However, this technique has limitations. In many cases, the energy required to capture the His bundle is significantly higher compared to standard right ventricular pacing, potentially leading to rapid battery depletion—particularly in patients with intra- or infra-Hisian block or distal left bundle branch block. Moreover, the intrinsic ventricular activity measured by the device (R wave) may be of very low amplitude at the His bundle position, making pacemaker programming difficult (13,14). As an alternative to His bundle pacing, in 2017, Huang et al. described the first case of direct left bundle branch pacing in patients who were ineligible for His bundle pacing. In this approach, the right ventricular lead (usually the same used for His bundle pacing) is deeply fixed in the interventricular septum, reaching the subendocardial region of the left ventricle, enabling direct stimulation of the His-Purkinje system through activation of the left bundle branch (11).

Academic centers worldwide have reported success with conduction system pacing techniques compared to traditional procedures. However, data analysis for this technique in the Brazilian population is scarce. Cardiac resynchronization through physiological pacing—whether by His bundle or left bundle branch lead implantation—offers more physiological stimulation and substantially lower procedural and equipment costs, making it a promising alternative to biventricular CRT. Initial studies comparing conduction system pacing with biventricular CRT are underway in several countries (Table 1). However, to date, no conduction system pacing study has had primary clinical endpoints such as mortality, hospitalization, or urgent heart failure visits.

Table 1. Summary of ongoing randomized clinical trials comparing conduction system pacing vs biventricular pacing.

Study	Population	Sample size	Follow-up (months)	Country	Primary Outcome
Left Bundle Branch Pacing Versus Biventricular Pacing for Cardiac Resynchronization Therapy **(LBBP-RESYNC)** (NCT04110431)	Symptomatic HF LVEF ≤40% LBBB	40	6	China	Difference in LVEF change between groups
LEft VEntricuLar Activation Time Shortening With Physiological Pacing vs Biventricular Resynchronization Therapy (**LEVEL-AT**) (NCT04054895)	Symptomatic HF LVEF ≤35% LBBB	70	12	Spain	Change in left ventricular activation time (ms)
Acute Hemodynamic Effects of Pacing the His Bundle in Heart Failure (**HEPA-His**) (NCT04701112)	Symptomatic HF LBBB	30	0 (acute)	Sweden	Change in stroke volume (ml) at rest
HIS-Purkinje Conduction System Pacing Optimized Trial of Cardiac Resynchronization Therapy (**HOT-CRT**) (NCT04561778)	Symptomatic HF LVEF <50%	100	6	USA	Change in LVEF (%); freedom from major complications
Effect of His Bundle Pacing in Treatment of Slow Arrhythmia on Function of Left Atrial and Ventricle (NCT03590353)	Bradyarrhythmia	84	12	China	Immediate and late electrophysiological parameters

Abbreviations: HF – Heart Failure, LBBB – Left Bundle Branch Block, LVEF – Left Ventricular Ejection Fraction.

RATIONALE

Conduction system pacing techniques have demonstrated procedural feasibility, reduced device-related costs, and clinical outcomes comparable to biventricular cardiac resynchronization therapy (CRT) in prior studies. However, there is a lack of prospective data evaluating their effectiveness using clinically meaningful primary endpoints and health economic impact within the Brazilian public health system (15–20).

2. OBJECTIVES

2.1 Primary Objective

To assess whether conduction system pacing is non-inferior to biventricular cardiac resynchronization therapy in terms of heart failure–related clinical outcomes over a 12-month follow-up period.

2.2 Conceptual Hypothesis

In patients with congestive heart failure and systolic disfunction eligible for cardiac resynchronization therapy, conduction system pacing is non-inferior to biventricular pacing regarding heart failure–related clinical outcomes.

2.3 Secondary Objectives

The main secondary objective is to assess whether the superiority of conduction system pacing (CSP) is superior to biventricular pacing for the economic endpoint, defined as total direct medical cost. Additional secondary endpoints, evaluated over a 12-month follow-up period, aim to compare both treatment arms regarding: time to first event (composite of all-cause mortality, heart failure hospitalization, or urgent heart failure visits); change in left ventricular ejection fraction (LVEF); change in left ventricular end-diastolic volume; change in Kansas City Cardiomyopathy Questionnaire (KCCQ) Overall Summary Score (OSS);

change in New York Heart Association (NYHA) functional class; change in six-minute walk test (6MWT) distance; change in B-type Natriuretic Peptide (BNP) and N-terminal pro–B-type Natriuretic Peptide (NT-proBNP) levels; change in EuroQol-5D (EQ-5D) score; change in QRS duration; and a hierarchical composite endpoint including death, heart failure hospitalization, urgent HF visits, and change in KCCQ Clinical Summary Score (CSS).

3. STUDY DESIGN

3.1 Design

This is a randomized, controlled, multicenter, non-inferiority clinical trial. The study is single-blinded (participant-blinded) with independent, blinded adjudication of outcomes.

3.2 Eligibility

3.2.1 Site Eligibility Criteria

Participating sites must be public or private hospitals in Brazil equipped to perform both conduction system pacing and biventricular pacing for cardiac resynchronization therapy. Preference will be given to institutions that serve the public healthcare system and provide outpatient care to patients at high cardiovascular risk. To qualify for this study, sites must meet the following criteria: (i) have an operational electrophysiology laboratory with appropriate infrastructure, including a recording system; (ii) employ at least one electrophysiologist with certified experience in cardiac resynchronization therapy; and (iii) submit a completed site feasibility questionnaire.

3.2.2 Participant Eligibility Criteria

3.2.2.1 Inclusion Criteria:

- Men and women aged ≥18 years
- Symptomatic congestive heart failure (NYHA class II–III) of either ischemic or non-ischemic etiologies

- Left ventricular ejection fraction (LVEF) ≤35%
- Presence of left bundle branch block (LBBB) on electrocardiogram with QRS duration ≥130 ms
- Clinical indication for cardiac resynchronization therapy (CRT), as determined by the treating physician
- Clinically stable, as assessed by the treating physician
- Receiving maximally tolerated doses of angiotensin-converting enzyme inhibitors (ACEi), angiotensin receptor blockers (ARB), or angiotensin receptor–neprilysin inhibitors (ARNI); beta-blockers; and mineralocorticoid receptor antagonists

3.2.2.2 Exclusion Criteria:

- Heart failure classified as NYHA Class IV
- Life expectancy less than 12 months due to any condition
- Dementia or advanced cerebrovascular disease
- Planned to receive an implantable cardioverter-defibrillator (ICD) alone or with CRT
- Concurrent participation in another clinical trial involving cardiac pacing
- Pregnant women or women of childbearing potential (pre-menopausal not using contraception)
- Inability to understand or sign the informed consent form

3.3 Outcomes

3.3.1 Primary Outcome

The primary outcome, defined as the "heart failure-related net composite outcome," is an ordinal scale endpoint assessed between groups using a proportional odds model. The scale is hierarchically structured as follows: (1) all-cause mortality; (2) heart failure hospitalization; (3) urgent visit for heart failure with a length of stay <24 hours; and (4) absolute change in LVEF from baseline to follow-up at up to 12 months, categorized into 5% intervals (e.g., …,

(-10, -5], (-5, 0], (0, +5], (+5, +10], …), with the highest improvement in LVEF represented by the final category. Non-inferiority of conduction system pacing compared to biventricular pacing will be assessed using a non-inferiority margin corresponding to an upper bound of the 95% confidence interval for the odds ratio <1.2. If non-inferiority is confirmed, superiority will be tested preserving the alpha and using the same methodology.

3.3.2 Secondary Outcomes

The main secondary outcome is the total direct medical cost associated with CSP compared with biventricular pacing over a 12-month period.

Other secondary outcomes, assessed up to 12 months, include:

- Time to the first event of all-cause mortality, heart failure hospitalization, or urgent visit for heart failure
- LVEF
- Left ventricular end-diastolic volume
- KCCQ-OSS
- NYHA functional class
- 6MWT
- BNP or NT-proBNP levels
- EQ5D
- QRS complex duration
- Hierarchical composite outcome including all-cause mortality, heart failure hospitalization, urgent visit for heart failure, and change in KCCQ-CSS

3.3.3 Exploratory Outcomes

Exploratory outcomes, assessed up to 12 months, include:

- Left ventricular end-systolic volume

- Proportion of patients achieving KCCQ-OSS improvements of ≥5, ≥10, and ≥20 points
- Duration of the index procedure
- Index procedure complication rate
- Implant-related adverse events
- Cost-effectiveness
- Predictors of cost
- Budget impact analysis
- Outcomes assessed via cardiopulmonary exercise testing (CPET):
  - Peak VO_2 (primary CPET endpoint)
  - Percent predicted peak VO_2
  - VE/VCO_2 slope
  - OUES
  - Resting $PETCO_2$
  - Heart rate recovery at 1 minute
  - Time to 50% heart rate recovery (T ½)

The CPET substudy will be conducted at selected participating sites equipped with the necessary infrastructure and qualified personnel to perform cardiopulmonary exercise testing.

3.3.4 Safety Outcomes

Safety outcomes include variables monitored after the conduction of CRT procedures, both at the time of implantation and throughout the follow-up period until study completion. These outcomes are collected through in-person or telephone interviews conducted by investigators at participating sites and recorded in the case report form (CRF). Adverse events (AEs) are classified into three categories:

1) *Procedure-Related Adverse Events*

These include complications occurring during the implantation procedure, such as pneumothorax, myocardial perforation, cardiac tamponade, or hemorrhage. Although rare, these events require immediate recognition and management during the hospitalization for the index procedure.

2) *Device-Related Adverse Events*

These refer to complications associated with the implanted device, such as lead dislodgement, lead fracture, elevated pacing thresholds that prevent effective stimulation, or diaphragmatic (phrenic nerve) stimulation, among others.

3) *Adverse Events Unrelated to the Procedure or Device*

These include any adverse events not fitting the definitions above.

3.3.5 Adjudicated Outcomes

The following expected outcomes will be reported and submitted for central adjudication:

- all deaths, to determine the cause of death;
- all hospitalization events, to determine whether they were caused by heart failure;
- all urgent visit events, to determine whether they were caused by heart failure.

Events will be adjudicated based on the criteria detailed in the event adjudication protocol. Additionally, all echocardiograms (baseline, 6-month, and 12-month) will be submitted to an independent and blinded cardiovascular imaging core laboratory for blinded assessment.

3.3.6 Adverse Events

An adverse event (AE) is defined as any undesirable medical occurrence in a clinical

trial participant who has received a pharmaceutical product, including an exacerbation of a pre-existing condition. The event does not necessarily need to have a causal relationship with the treatment under investigation.

Serious Adverse Events (SAE)

All serious adverse events must be reported, regardless of their relation to the study intervention. Components of the primary endpoint—death, hospitalization, or urgent heart failure visit events—that meet the criteria for an SAE will be reported as study endpoints rather than as SAEs.

A serious adverse event is defined as any AE that results in death, is life-threatening, results in persistent or significant disability/incapacity, requires or prolongs hospitalization, results in a congenital anomaly or birth defect, or is otherwise considered serious based on appropriate medical judgment. This includes events that may not meet the above criteria but may jeopardize the patient and require medical or surgical intervention to prevent one of the listed outcomes.

The intensity of an AE should be assessed as follows:

- **Mild:** Awareness of signs or symptoms that are easily tolerated.
- **Moderate:** Discomfort sufficient to interfere with usual activities.
- **Severe:** Incapacitating or preventing the performance of normal activities or work.

Causality Assessment

Causality must be assessed using clinical judgment, considering all relevant factors, including the nature and timing of the event in relation to the investigational device. The PhysioSync-HF Project Office will report SAEs to ANVISA (Brazilian Health Regulatory Agency) in a timely manner, as per the Serious Adverse Event Management Plan.

3.4 Randomization and Allocation Concealment

Patients will be randomly assigned in a 1:1 ratio to either CRT strategy. The randomization list generated by a *blockrand* function, which generates random assignments. The probability of allocation to either CRT group will be equal, and stratification will be performed by center using variable block sizes of 4 and 6 patients. Each CRT arm will be assigned a numerical code, and only these codes will be used during the allocation process, which will be managed electronically by the coordinating center (Hospital Moinhos de Vento) to ensure allocation concealment. The coordinating center will notify participating sites of the assigned intervention following randomization through REDCap interface hosted at HMV.

3.5 Blinding

As the interventions inherently require distinct procedural approaches, treatment allocation will be open to investigators. However, in addition to blinding to trial participants, all clinical outcomes will be independently adjudicated by a blinded independent Clinical Events Committee (CEC). Unblinding will occur only after the last patient concludes follow-up and database lock has occurred, or in the setting of safety concerns related to the CRT procedures, or other exceptional circumstances requiring a change in patient management as determined by an Independent Data and Safety Monitoring Board. This committee will consist of cardiologists with expertise in heart failure and electrophysiology, responsible for early safety surveillance throughout the study, supported by a clinical events adjudication team, clinical care teams, and patients.

3.6 Study Procedures

Hospitals with invasive electrophysiology services across the five geographic regions of Brazil were invited to participate. Twenty centers were contacted, and only those with signed Institutional Cooperation Agreements and complete regulatory documentation were included in the Plataforma Brasil, the National Council for Scientific and Technological Development platform for Institutional Review Board assessments. All participating centers received

standardized training in study protocol implementation.

Before assessing eligibility criteria, patients must be evaluated for underlying heart failure with an indication for CRT, confirmed by electrocardiogram and imaging. Eligible patients must have been clinically stable for at least three months and receiving optimized guideline-directed medical therapy for heart failure, defined as maximally tolerated doses of the following medication classes for at least three months: ACE inhibitors or angiotensin receptor blockers (ARBs) or angiotensin receptor–neprilysin inhibitors (ARNIs), beta-blockers, and mineralocorticoid receptor antagonists.

After providing informed consent at the specialized HUB center, participants will undergo baseline assessments including electrocardiogram (EKG), echocardiogram, B-type natriuretic peptide (BNP or NT-proBNP), six-minute walk test (6MWT), and quality of life and functional capacity questionnaires.

Quality of life is assessed using two validated instruments: the EuroQol 5-Dimensions (EQ-5D) and the Kansas City Cardiomyopathy Questionnaire (KCCQ). The EQ-5D, validated for the Brazilian population and in electronic format, evaluates mobility, self-care, usual activities, pain/discomfort, and anxiety/depression. The KCCQ is a 23-item, self-administered questionnaire that assesses patients' perception of their health status across six domains: symptoms, physical function, quality of life, social limitation, symptom stability, and self-efficacy.

The 6MWT requires a hallway with marked distances and trained staff. Patients are instructed to walk at their own pace on a flat surface for six minutes, aiming to cover the longest possible distance. The test is discontinued in cases of limiting symptoms such as fatigue, chest pain, dyspnea, pallor, leg cramps, sweating, or claudication.

Cardiopulmonary exercise testing (CPET) is to be performed at baseline and at 6- and 12-month follow-up visits in centers equipped and qualified for this assessment. The ramp protocol used is tailored to the reduced physical capacity of this population. CPET with

expired gas analysis is conducted on a treadmill with the following parameters:

- Speed: 0–22 km/h; grade: 0–26%;
- Initial speed: 2.0 or 2.5 km/h; initial grade: 0%;
- Increments: 0.5 km/h per minute and 1% grade per minute, targeting peak exertion within 8–12 minutes.

Patients should be randomized as soon as possible after eligibility confirmation, minimizing the interval between randomization and the procedure, while accounting for each center's logistics. Considering the clinical benefits of CRT in mortality reduction and NYHA functional class improvement, centers are encouraged to perform the intervention as soon as possible, according to the institutional availability and patient-specific needs.

In general, participating centers should admit patients for hospital-based CRT implantation, with post-procedure monitoring for at least 6 hours to detect potential acute complications. Biventricular CRT is a well-established procedure routinely performed in electrophysiology centers, and technical proficiency in its execution was a prerequisite for center inclusion in the study. CRT via conduction system pacing must adhere to a previously published protocol developed by the study's steering committee (21), with supervision from the study's coordinating center. A version of the following intervention protocol will be provided to all participating centers.

3.6.1 His-Purkinje Conduction System Pacing Protocol

I The initial approach to conduction system pacing involves implantation of the lead in the left bundle branch (LBB). Successful LBB pacing is defined by the presence of a characteristic electrocardiographic pattern in lead V1 (qR or rqR') along with at least one of the following criteria:

- Recording of an LBB/Purkinje potential preceding the QRS complex, with an interval between the local potential and surface ECG of 10–50 ms;

- Maintenance of left ventricular activation time (LVAT) < 90 ms during unipolar pacing at both high and low output;
- Gradual transition between non-selective and selective LBB capture patterns during continuous unipolar pacing at varying pacing output;
- Shift from a non-selective capture pattern to a septal myocardial capture pattern near the final capture threshold;
- Response to extra stimulus testing showing either pure myocardial capture or selective LBB capture.

II If optimal LBB pacing cannot be achieved, the lead should be implanted at the His bundle. His bundle pacing is considered unsuccessful if intraoperative parameters demonstrate a pacing threshold > 2 V at 1 ms pulse width and/or an R wave amplitude < 2 mV.

III In the event of failure to implant the lead in both the LBB and His bundle locations, deep septal pacing of the left ventricle should be performed.

IV When the conduction block is not adequately corrected by septal pacing, implantation of a complementary lead in the coronary sinus is permitted.

It is important to note that if effective resynchronization cannot be achieved during the procedure, patients originally randomized to the biventricular CRT arm will be automatically crossed over to the conduction system pacing arm, and vice versa (i.e., crossover is allowed). However, in the primary analysis, patients will be analyzed according to their original randomization group. Hospital discharge may be considered at least 6 hours post-procedure (or according to each center's established routine) if no clinical complications arise and the attending physician deems it safe.

3.6.2 Follow-up

Following CRT, all patients must undergo an in-person evaluation approximately 30 days (±5 days) post-intervention to assess pacemaker parameters (QRS complex, capture threshold, left ventricular activation time, impedance, and R wave amplitude) and to evaluate for potential late complications related to the procedure. The purpose of this initial visit is to confirm that participants remain clinically stable and to exclude lead dislodgements.

Subsequent follow-up visits are scheduled at 6 months (±20 days) and 12 months (±20 days) post-CRT at the participating sites. During these visits, patients undergo clinical evaluation, laboratory testing (BNP or NT-proBNP) and echocardiography to assess changes in left ventricular ejection fraction (LVEF), end-systolic volume, end-diastolic volume, end-diastolic diameter, and end-systolic diameter. The follow-up timeline will be calculated from the date of the index procedure.

3.6.3 Description of Study Visits and Assessments

A detailed summary of study procedures by visit is presented in **Figure 2.**

Figure 2. Schematic overview of the PhysioSync-HF study protocol

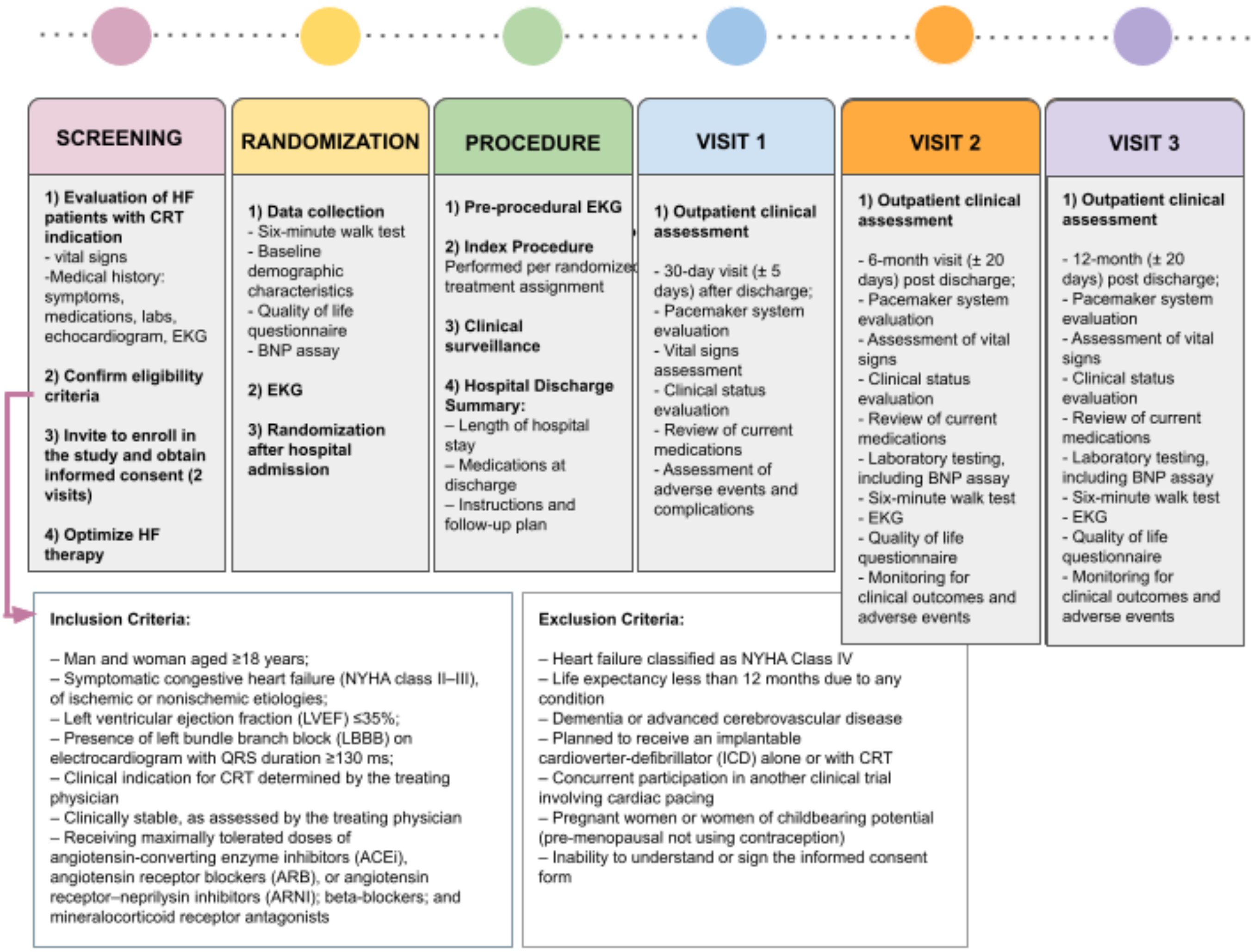

3.6.4 Handling of Patients Incorrectly Enrolled or Randomized

In cases of inadequate inclusion of a patient with HF (due to errors in eligibility criteria), the participating center must contact the coordinating center, which will determine whether the patient should continue or be withdrawn from the study—provided this occurs before randomization. After randomization, even if performed incorrectly, participants must remain in the study and continue clinical follow-up.

3.6.5 Data Collection for Economic Analysis

Resource utilization for cost analysis will be measured using a bottom-up approach, tracking participants throughout the clinical trial with separate methods to estimate costs for 1) index hospitalization and 2) post-discharge follow-up.

For estimating costs related to the index hospitalization, a microcosting study based on Time-Driven Activity-Based Costing (TDABC) will be conducted. This methodology will be applied through the following steps:

1) mapping the care pathway and identifying key activities undergone by the patient;

2) identifying all resources and departments utilized during the care pathway;

3) estimating the total cost of each resource identified;

4) estimating the hourly capacity of each resource or department and calculating the cost capacity rate (CCR [BRL R$/hour]);

5) analyzing the time each resource is used per patient, integrating time data collection into the CRF related to the index hospitalization;

6) calculating the total cost per patient during the index hospitalization.

For cost estimates related to healthcare utilization during post-discharge follow-up, a macro-costing approach will be applied from the perspective of the Brazilian health system, and cost estimates will be assigned to urgent visits and hospitalizations for decompensated heart failure. Cost estimates will be extracted from the Brazilian Unified Health System Procedures, Medications, and Other Inputs Table (SIGTAP), consistent with prior publications. The Brazilian Hierarchical Classification of Medical Procedures (CBHPM), a reference for costs in the private healthcare system, may also be utilized. For outpatient management costs for heart failure, micro-costing estimates from prior studies employing TDABC will be used, stratified according to NYHA functional class at baseline and at 12 months.

Finally, for the exploratory budget impact analysis, cost data will be integrated with

epidemiological estimates of the eligible population size from the perspective of the Brazilian Unified Health System. The budget impact analysis will evaluate the total cost for each CRT arm under study from the healthcare system perspective, considering all potentially eligible patients.

3.7 Concomitant Therapies

The Physio Sync-HF trial will not provide specific recommendations regarding predefined concomitant therapies during the index cardiac resynchronization therapy (CRT) procedure. Participating sites (HUBs) will be instructed to provide standard care in accordance with their institutional practices, while ensuring that patient management adheres to current evidence-based guidelines for the optimal treatment of heart failure, both at the time of enrollment and for pre-existing conditions. Healthcare teams at each HUB will be instructed to counsel patients at the time of hospital discharge on adopting healthy lifestyle habits, including regular physical activity, a balanced diet, and adherence to prescribed pharmacological therapy.

3.8 Risks and Benefits to Patients

Based on current heart failure treatment guidelines, eligible patients in this study have a formal indication for CRT, with established benefits in mortality reduction and decreased heart failure-related hospitalizations. Both study arms will receive appropriate treatment without bias. Since the study is primarily conducted in public hospitals, PhysioSync-HF has the potential to reduce waiting lists within the Brazilian Unified Health System (SUS) for patients awaiting CRT.

The primary risks are related to adverse events inherent to the intervention itself, including perioperative complications such as vascular puncture, anesthesia-related issues, and surgical site infection. To mitigate these risks, only patients with formal indications for CRT, as per the Brazilian Society of Cardiology guidelines (22) and the Ministry of Health, and in

whom the expected benefit outweighs the procedural risk, will be included. Patients will be informed of these potential adverse events, along with their therapeutic and prophylactic management. Any adverse events occurring must be documented in the Case Report Form (CRF) and managed by the medical teams at participating centers.

3.9 Risks and Benefits to the Brazilian Unified Health System (SUS)

This project addresses key challenges faced by the Brazil's public health system (SUS). The proposed intervention requires trained teams capable of performing CRT implantations; therefore, the study includes a component of capacity building to train staff at participating centers, ensure high-quality procedures, and prioritize patient safety. The national collaboration established through this project may serve as a foundation for future research initiatives. Potential benefits to the SUS include validating the safety and efficacy of a modern CRT approach with the potential to significantly reduce procedural costs; expanding access and coverage for patients across Brazil; and improving the quality of life for individuals with heart failure.

3.10 Follow-up

Patients will be followed for a total of 12 months after the procedure, with scheduled in-person visits at enrollment/screening and at 1, 6, and 12 months post-procedure. If patients are unable to attend scheduled visits in person, remote contact via telephone or video call by local study teams will be arranged.

3.11 Loss to Follow-up

For patients who miss scheduled follow-up visits and do not respond to phone calls or emails—and have not explicitly expressed a desire to withdraw from the study—active outreach efforts will be made. These will include contacting family members or individuals listed in the study enrollment documents and informed consent form of the PhysioSync-HF

study. Patients will be encouraged to remain in the study, and investigators at each HUB will actively support participants to remain engaged in the study. In cases where patients choose to voluntarily withdraw, this will be documented in the CRF, and the patient will be advised to continue their clinical care at their HUB or designated referral center.

If pregnancy is confirmed during the follow-up period, the patient's data will still be recorded in the CRF and included in all subsequent follow-up visits and analyses.

3.12 Sample Size Calculation

The one-year mortality incidence was estimated at 6.24% for both groups, based on the RAFT study, which reported 20.8% mortality over 40 months (calculated by simple proportional scaling). Hospitalization due to heart failure was estimated at 5.85% at one year for both groups (RAFT study: 19.5% hospitalizations over 40 months). Urgent heart failure visits were estimated at 0.5% per year in both groups, based on the DAPA-HF study reporting 0.5 events per 100 patient-years. An absolute increase in left ventricular ejection fraction (LVEF) of 16% was assumed for the intervention group and 14% for the control group, a conservative estimate drawn from the His-Alternative study, which observed increases of 16% and 13%, respectively, with a standard deviation of 6.5 (values of 6 and 7 reported in His-Alternative). To achieve 80% power with a two-sided alpha of 5% (one-sided alpha of 2.5%) and a non-inferiority margin of OR < 1.2 (upper confidence interval limit), and accounting for 5% non-informative losses, a total sample size of 304 patients was required for study completion.

Considering slower-than-expected patient enrollment and results from a meta-analysis published during the course of PhysioSync-HF (Gin et al., Heart Rhythm 2023) indicating a difference in LVEF increase between 2.70% and 5.18% favoring the intervention group—greater than the original 2% estimate—the Steering Committee opted to recalculate the sample size (24). The revised calculation assumed a 3% difference in LVEF increase favoring the

intervention group. This difference was consistent with the originally anticipated effect from the His-Alternative study but was previously conservatively estimated at 2% due to limited data. With this update, the required sample size decreased from 304 to 180 patients, maintaining the non-inferiority margin of OR <1.2 and 80% power.

3.13 Statistical Analysis Plan

Baseline characteristics of the study population will be presented by group and for the total sample. Quantitative variables will be described using mean, standard deviation, median, 25th and 75th percentiles, and the number of valid observations. Qualitative variables will be summarized by absolute frequencies (number of patients) and relative frequencies (percentages). The primary outcome analysis will use the modified intention-to-treat population, including all randomized patients who initiated the procedure. The proportional odds assumption will be tested using appropriate methods. If non-inferiority of the primary outcome is established, superiority testing will follow while preserving the alpha level. The main secondary outcome will be the analysis of total direct medical costs. Additionally, a Cox regression will be performed to estimate the hazard ratio (HR) and 95% confidence interval (CI) for a composite endpoint comprising the binary components of the primary outcome (all-cause mortality, heart failure hospitalization, urgent heart failure visit). Kaplan-Meier curves will estimate time to first event. Additional survival analyses will be conducted as sensitivity analyses if warranted. Statistical significance will be set at a two-sided 5% level for all analyses, including the primary non-inferiority outcome (one-sided alpha of 2.5%). A detailed Statistical Analysis Plan is available as a separate document. Data analysis will be conducted by the coordinating center using R version 3.6.2 (or higher).

3.14 Data Collection System

Data will be collected via electronic case report forms through the REDCap system

over the internet. Site teams will enter data directly into the system. All forms will be electronically signed by the principal investigator or authorized personnel at each center. Training and support for system use will be provided by the coordinating center.

All enrolled patients will have their clinical and study-related data collected by qualified personnel trained in confidentiality and secure data storage. Data for participants who withdraw during follow-up will be retained for the period from inclusion until withdrawal. Investigators at participating centers will maintain data confidentiality and control access to the electronic platform, protecting privacy in accordance with local regulatory requirements.

To ensure the confidentiality and handling of sensitive personal data, as defined by Brazilian Law No. 13,709 of August 14, 2018, all data will be stored in encrypted form, accessible only to the study team at the coordinating center. Interviewers will not have access to consolidated data and will be trained to maintain confidentiality and anonymity, as well as to properly handle, transport, and archive signed informed consent forms. Only responsible researchers will have access to patient identifiers.

3.15 Recruitment

Strategies to enhance recruitment include but are not limited to: listing potentially eligible patients by center; active screening in heart failure outpatient clinics; evaluating eligibility of all patients planned for CRT implantation; maintaining active communication between the coordinating and participating centers to resolve queries; and continuous monitoring to identify centers with suboptimal recruitment, allowing timely corrective actions.

4. ETHICAL CONSIDERATIONS AND GOOD CLINICAL PRACTICE

The Physio Sync-HF study will be conducted in accordance with the ethical principles outlined in both international and national guidelines for research involving human subjects. The study protocol and informed consent form will be submitted to the Institutional Review

Board (IRB) at Hospital Moinhos de Vento, as well as to the IRBs of all participating sites. Study activities will commence only after all required approvals have been obtained. The informed consent form was developed based on Resolution No. 466/12 of the Brazilian National Health Council.

The study protocol will be submitted for ethical review by the Hospital Moinhos de Vento IRB, the sponsoring institution. Each participating center will conduct its own ethical review through the Plataforma Brasil system, under the "participating site" designation. Prior to submission for ethical review, the participating institution must obtain authorization from its administrative leadership to conduct the research.

4.1 Local Study Approval

Prior to study initiation at each participating site, the protocol must receive approval from that site's Institutional Review Board, regardless of any prior approval granted by the coordinating center's IRB.

4.2 Informed Consent

All patients selected for participation in the study must sign the Informed Consent Form (ICF) prior to undergoing any study-related procedures.

4.3 Central Study Approval

Prior to study initiation, the protocol, site-specific informed consent forms, and any other required documents must be submitted to and approved by the Hospital Moinhos de Vento IRB, in accordance with local regulatory requirements. The protocol will also be registered on ClinicalTrials.gov.

5. STUDY COORDINATION

5.1 Coordinating Center

The Coordinating Center for the PhysioSync-HF study is Moinhos de Vento Hospital, an institution with extensive experience in conducting clinical research. The coordinating team is responsible for providing training, guidance, and support to participating centers to ensure adherence to the study protocol. This team has the necessary expertise in research methodology and biostatistics and is supported by award-winning career researchers.

5.2 Steering Committee

The Steering Committee members of the PhysioSync-HF study oversee the clinical trial, including decisions to suspend or modify study procedures if necessary, managing challenges related to protocol implementation, reviewing and interpreting data, and preparing the final manuscript. The Committee is chaired by a designated chairperson. Coordination is conducted via in-person or videoconference meetings held at least quarterly. All other study committees report directly to the Steering Committee.

5.3 Executive Committee

Composed of members of the Steering Committee affiliated with the Coordinating Center, the Executive Committee functions as the administrative and operational arm of the Steering Committee, responsible for operational decisions on its behalf.

5.4 Publication Committee

Members of the Executive Committee are selected to form a Publication Committee tasked with drafting and submitting the final manuscript for publication.

5.5 Adjudication Process

The Clinical Events Committee (CEC) is responsible for the adjudication of all clinical events. Potential endpoint events will be entered into the CEC tracking database and undergo

administrative review to ensure that all required documentation is available. The adjudication process will rely on original source documents, such as official medical reports, signed diagnostic tests, and other relevant clinical records. Imaging DVDs or digital records are not required unless official reports are unavailable, or discrepancies arise between adjudicators or between clinical presentation and test results.

The research coordinator will compile the necessary documents from the electronic case report form (eCRF) into a CEC adjudication packet. Two copies of each event packet will be sent by the coordinating center (HMV) to the CEC, where they will be randomly assigned to two independent medical reviewers. Each reviewer will independently assess and document their findings within the adjudication packet. Agreement between both reviewers will finalize the adjudication. In case of disagreement between reviewers, or if upon reviewer's request, a third reviewer will assess the case to determine the final outcome. Final adjudications will be entered into the database by the CEC coordinator. Signed adjudication forms will be archived in the CEC files. Further procedural details will be maintained in a separate document at HMV.

All adjudications will be documented based on predefined diagnostic criteria. For precedent-setting cases, the CEC chair will document the adjudication in detail, and the case will serve as a reference for future consistency in endpoint definitions.

5.6 Data Quality Management

Data quality is ensured through multiple procedures, including:

1) Mandatory training sessions for all investigators before study initiation to standardize procedures, including data collection;
2) Supervision of the first conduction system stimulation intervention at each center by the study's electrophysiology coordinator to ensure procedural quality criteria;
3) Investigators may contact the Coordinating Center to resolve any issues that arise;
4) Data entry via the Moinhos de Vento Hospital Data Management System is subject to checks for missing fields, plausible and valid value ranges, and logical consistency. Investigators receive immediate notifications of data entry errors;

5) Statistical methods to identify inconsistencies are applied approximately every two weeks, with centers notified to correct issues;
6) Fraud detection analyses are conducted every 90 days;
7) Monitoring visits are performed at participating centers during the study;
8) The Coordinating Center reviews monthly detailed reports on screening, enrollment, follow-up, data consistency, and completeness, initiating corrective actions as needed.

5.7 Independent Data and Safety Monitoring Board

The Independent Data and Safety Monitoring Board (DSMB) for PhysioSync-HF comprises:

a. A clinician experienced in heart failure;
b. A clinician experienced in electrophysiology;
c. A researcher, epidemiologist, or statistician with expertise in randomized trial analysis.

One member, selected by the Steering Committee, will serve as Chair. The Chair must be a senior cardiology researcher holding a leadership position at a reputable institution.

The DSMB meets at least biannually to assess study status, recruitment rates, protocol adherence, data quality, loss to follow-up, site-specific issues, and other relevant concerns.

No interim analyses for futility or overwhelming efficacy are planned. The DSMB may recommend study termination for safety reasons based on its evaluation.

5.8 Sponsor Responsibilities

The study's objectives were designed solely to advance scientific knowledge applicable to clinical practice, free from conflicts of interest. Financial support sources will be acknowledged in presentations and publications but will have no influence over publication decisions or content. PhysioSync-HF results will be published regardless of positive or negative outcomes.

5.9 Responsibilities of Investigators and Subinvestigators

The principal investigator (PI) at each center oversees daily study operations at their site, assisted by subinvestigators and research coordinators. Most tasks may be delegated to qualified team members listed on delegation logs; however, the PI retains ultimate legal responsibility. Investigators are accountable for all legal and ethical study requirements, protocol adherence, and ensuring data quality and accuracy.

5.10 Monitoring

Authorized representatives of the PhysioSync-HF project office will conduct periodic site visits to review data quality and study integrity. They will examine all study records, cross-check with source documents, discuss study conduct with investigators, and verify that facilities remain adequate.

5.11 Publication of Results

The success of PhysioSync-HF depends on the collaborative efforts of investigators, coordinators, and patients. Primary results will be published under the authorship of the Study Steering Committee. The names of PhysioSync-HF investigators will be listed at the end of the manuscript. Up to three members per research team involved in each study phase may be included as co-authors at the discretion of the PI and Publication Committee. Individual authorship decisions will be made by the Publication Committee.

5.12 Protocol Amendments

Any protocol amendments must be documented in writing and signed by the Principal Investigator. Approval by the relevant Ethics Committees is required prior to implementation, unless immediate safety concerns necessitate urgent changes.

6. REFERENCES

1. Kandala J, Upadhyay GA, Altman RK, Parks KA, Orencole M, Mela T, et al. QRS morphology, left ventricular lead location, and clinical outcome in patients receiving cardiac resynchronization therapy. Eur Heart J. agosto de 2013;34(29):2252–62.

2. McAlister FA, Ezekowitz J, Hooton N, Vandermeer B, Spooner C, Dryden DM, et al. Cardiac resynchronization therapy for patients with left ventricular systolic dysfunction: a systematic review. JAMA. 13 de junho de 2007;297(22):2502–14.

3. Cleland JGF, Daubert J-C, Erdmann E, Freemantle N, Gras D, Kappenberger L, et al. The effect of cardiac resynchronization on morbidity and mortality in heart failure. N Engl J Med. 14 de abril de 2005;352(15):1539–49.

4. Moss AJ, Hall WJ, Cannom DS, Klein H, Brown MW, Daubert JP, et al. Cardiac-Resynchronization Therapy for the Prevention of Heart-Failure Events. N Engl J Med. 1º de outubro de 2009;361(14):1329–38.

5. Chinitz JS, d'Avila A, Goldman M, Reddy V, Dukkipati S. Cardiac resynchronization therapy: who benefits? Ann Glob Health. fevereiro de 2014;80(1):61–8.

6. Rohde LE, Bertoldi EG, Goldraich L, Polanczyk CA. Cost-effectiveness of heart failure therapies. Nat Rev Cardiol. junho de 2013;10(6):338–54.

7. Gazzoni GF, Fraga MB, Ferrari ADL, Soliz P da C, Borges AP, Bartholomay E, et al. Preditores de Mortalidade Total e de Resposta Ecocardiográfica à Terapia de Ressincronização Cardíaca: Um Estudo de Coorte. Arq Bras Cardiol. dezembro de 2017;109(6):569–78.

8. Daubert C, Behar N, Martins RP, Mabo P, Leclercq C. Avoiding non-responders to cardiac resynchronization therapy: a practical guide. Eur Heart J. 14 de maio de 2017;38(19):1463–72.

9. Ajijola OA, Upadhyay GA, Macias C, Shivkumar K, Tung R. Permanent His-bundle pacing for cardiac resynchronization therapy: Initial feasibility study in lieu of left ventricular lead. Heart Rhythm. 2017;14(9):1353–61.

10. Huang W, Su L, Wu S, Xu L, Xiao F, Zhou X, et al. Long-term outcomes of His bundle pacing in patients with heart failure with left bundle branch block. Heart Br Card Soc. 2019;105(2):137–43.

11. Huang W, Su L, Wu S, Xu L, Xiao F, Zhou X, et al. A Novel Pacing Strategy With Low

and Stable Output: Pacing the Left Bundle Branch Immediately Beyond the Conduction Block. Can J Cardiol. 1º de dezembro de 2017;33(12):1736.e1-1736.e3.

12. Sharma PS, Dandamudi G, Herweg B, Wilson D, Singh R, Naperkowski A, et al. Permanent His-bundle pacing as an alternative to biventricular pacing for cardiac resynchronization therapy: A multicenter experience. Heart Rhythm. 2018;15(3):413–20.

13. Wang Songjie, Wu Shengjie, Xu Lei, Xiao Fangyi, Whinnett Zachary I., Vijayaraman Pugazhendhi, et al. Feasibility and Efficacy of His Bundle Pacing or Left Bundle Pacing Combined With Atrioventricular Node Ablation in Patients With Persistent Atrial Fibrillation and Implantable Cardioverter-Defibrillator Therapy. J Am Heart Assoc. 17 de dezembro de 2019;8(24):e014253.

14. Zanon F, Ellenbogen KA, Dandamudi G, Sharma PS, Huang W, Lustgarten DL, et al. Permanent His-bundle pacing: a systematic literature review and meta-analysis. Eur Eur Pacing Arrhythm Card Electrophysiol J Work Groups Card Pacing Arrhythm Card Cell Electrophysiol Eur Soc Cardiol. 01 de 2018;20(11):1819–26.

15. Upadhyay GA, Vijayaraman P, Nayak HM, Verma N, Dandamudi G, Sharma PS, et al. His Corrective Pacing or Biventricular Pacing for Cardiac Resynchronization in Heart Failure. J Am Coll Cardiol. 9 de julho de 2019;74(1):157–9.

16. Sharma PS, Dandamudi G, Naperkowski A, Oren JW, Storm RH, Ellenbogen KA, et al. Permanent His-bundle pacing is feasible, safe, and superior to right ventricular pacing in routine clinical practice. Heart Rhythm. 1º de fevereiro de 2015;12(2):305–12.

17. Lustgarten DL, Crespo EM, Arkhipova-Jenkins I, Lobel R, Winget J, Koehler J, et al. His-bundle pacing versus biventricular pacing in cardiac resynchronization therapy patients: A crossover design comparison. Heart Rhythm. julho de 2015;12(7):1548–57.

18. Upadhyay GA, Tung R. His Bundle Pacing for Cardiac Resynchronization. Card Electrophysiol Clin. setembro de 2018;10(3):511–7.

19. Qian Z, Zou F, Wang Y, Qiu Y, Chen X, Jiang H, et al. Permanent His bundle pacing in heart failure patients: A systematic review and meta-analysis. Pacing Clin Electrophysiol PACE. fevereiro de 2019;42(2):139–45.

20. Upadhyay GA, Vijayaraman P, Nayak HM, Verma N, Dandamudi G, Sharma PS, et al. On-treatment comparison between corrective His bundle pacing and biventricular pacing for

cardiac resynchronization: A secondary analysis of the His-SYNC Pilot Trial. Heart Rhythm. dezembro de 2019;16(12):1797–807.

21. Dal Forno ARJ, Ternes CMP, Rech JV, Nascimento HG, Lewandowski A, Damasceno G, d'Avila A. Estimulação do Ramo Esquerdo do Sistema His-Purkinje: Experiência Inicial. Arq Bras Cardiol. 2022; 118(2):505-516

22. Diretrizes Brasileiras de Dispositivos Cardíacos Eletrônicos Implantáveis (DCEI). Arq Bras Cardiol. dezembro de 2007;89(6):e210–37.

23. Kusumoto FM, Schoenfeld MH, Barrett C, Edgerton JR, Ellenbogen KA, Gold MR, et al. 2018 ACC/AHA/HRS Guideline on the Evaluation and Management of Patients With Bradycardia and Cardiac Conduction Delay: A Report of the American College of Cardiology/American Heart Association Task Force on Clinical Practice Guidelines and the Heart Rhythm Society. J Am Coll Cardiol. 20 de agosto de 2019;74(7):e51–1561.

24. Gin J, Chow CL, Voskoboinik A, Nalliah C, Wong C, Van Gaal W, Farouque O, et al. Improved Outcomes of Conduction System Pacing in Heart Failure with Reduced Ejection Fraction – A Systematic Review and Meta-analysis. Heart Rhythm. 2023;S1547527123022269..
