# Supplementary material for: Conduction System vs Biventricular Pacing in Heart Failure: The PhysioSync-HF Randomized Clinical Trial
Source: JAMA Cardiol. 2026 Mar 11;11(4):360–8. doi: 10.1001/jamacardio.2026.0101 (PMC12980360; doi:10.1001/jamacardio.2026.0101)
Supplement: Supplement 2. — Statistical Analysis Plan [file jamacardiol-e260101-s002.pdf]

Statistical Analysis Plan (SAP)

Conduction System Pacing Versus Biventricular Resynchronization in Patients with Chronic Heart Failure

PhysioSync-HF

ClinicalTrials.gov ID: NCT05572736

SAP Version: 1.0; April 22, 2025

Protocol Version: 8.0; November 21, 2024

This document is an English translation of the original Statistical Analysis Plan, which was written and applied in Portuguese.

Table of Contents

1. Background 5
2. Rationale 5
3. Objectives 6
   3.1. Primary Objective 6
   3.2. Secondary Objectives 6
4. Methods 6
   4.1. Design 6
   4.2. Randomization 6
   4.3. Eligibility 7
      4.3.1. Inclusion Criteria 7
      4.3.2. Exclusion Criteria 7
   4.4. Outcome Definitions and Measurements 7
      4.4.1. Primary Outcome 7
      4.4.2. Secondary Outcomes 8
      4.4.3. Exploratory Outcomes 8
      4.4.4. Safety Outcomes 9
      4.4.5. Adjudicated Outcomes 9
   4.5. Sample Size 9
   4.6. Interim Statistical Analyses 10
   4.7. Timing of Final Analysis 10
   4.8. Timing of Outcome Assessment 10
5. Analysis Populations 10

5.1. Intention-to-Treat (ITT) 10

5.2. Modified Intention-to-Treat (mITT) 10

5.3. Per Protocol (PP) 11

5.4. Safety Population (As-Treated) 11

6. Principles of Statistical Analysis 11

6.1. Confidence Intervals and p-values 11

7. Statistical Analysis 11

7.1. Screening 11

7.2. Follow-Up 11

7.3. Baseline Characteristics 13

7.4. Primary Outcome Analysis 13

7.5. Secondary Outcome Analysis 13

7.5.1. Economic Outcomes and Cost Analysis 13

7.5.2. Time to Death, Heart Failure Hospitalization, or Urgent HF Visit 14

7.6. Hierarchical Composite Outcome 14

7.7. Natriuretic Peptides 16

7.8 Functional Class 16

7.9. Other Secondary Outcomes 16

7.10 Sensitivity Analyses 18

7.11. Subgroup Analyses 18

7.12. Functional Capacity Sub-study 18

7.13. Missing Data 19

7.14. Additional Definitions 19

7.15. Statistical Software 19

8. References 20

Appendix A – Power Simulations 22

1. Background

Heart failure (HF) patients with limited response to Guideline-Directed Medical Therapy (GDMT) remain at high risk of morbidity, mortality, and adverse left ventricular remodeling (1,2). Randomized controlled trials (RCTs) have demonstrated a net clinical benefit of cardiac resynchronization therapy (CRT) in these patients, particularly in those with reduced ejection fraction and a wide QRS complex and left bundle branch block (LBBB) (3–5). However, 30–40% of the population receiving CRT fail to show clinical improvement with biventricular pacing, which is also a high-cost intervention from the Brazilian health system perspective (6–8).

As an alternative to conventional resynchronization—typically performed by implanting one lead in the right ventricular endocardium and a second lead in a coronary sinus branch—direct stimulation of the His-Purkinje system can be used, targeting either the His bundle or the left bundle branch (9–11). In His bundle pacing, the right ventricular lead is fixed near the apex of the Koch's triangle, allowing for selective or non-selective capture of the His-Purkinje system. By engaging the heart's native conduction system, this technique restores physiological ventricular activation and may prevent undesirable effects of conventional biventricular pacing, such as ventricular remodeling (12).

However, this technique has limitations. In many cases, the energy required to capture the His bundle is significantly higher compared to standard right ventricular pacing, potentially leading to rapid battery depletion—particularly in patients with intra- or infra-Hisian block or distal left bundle branch block. Moreover, the intrinsic ventricular activity measured by the device (R wave) may be of very low amplitude at the His bundle position, making pacemaker programming difficult (13,14). As an alternative to His bundle pacing, in 2017, Huang et al. described the first case of direct left bundle branch pacing in patients who were ineligible for His bundle pacing. In this approach, the right ventricular lead (usually the same used for His bundle pacing) is deeply fixed in the interventricular septum, reaching the subendocardial region of the left ventricle, enabling direct stimulation of the His-Purkinje system through activation of the left bundle branch (11).

Reference centers worldwide have reported success with conduction system pacing techniques compared to traditional procedures. However, data analysis for this technique in the Brazilian population is scarce. Cardiac resynchronization through physiological pacing—whether by His bundle or left bundle branch lead implantation—offers more physiological stimulation and substantially lower procedural and equipment costs, making it a promising alternative to biventricular CRT. Initial studies comparing conduction system pacing with biventricular cardiac resynchronization therapy (CRT) are underway in several countries. However, to date, no conduction system pacing study has had primary clinical endpoints such as mortality, hospitalization, or urgent heart failure visits.

2. Rationale

Conduction system pacing techniques have demonstrated procedural feasibility, reduced device-related costs, and clinical outcomes comparable to biventricular cardiac resynchronization therapy (CRT) in prior studies. However, there is a lack of prospective data evaluating their

effectiveness using clinically meaningful primary endpoints and health economic impact within the Brazilian health system (15–20).

3. Objectives

3.1. Primary Objective

To assess whether conduction system pacing is non-inferior to biventricular cardiac resynchronization therapy in terms of heart failure–related net clinical outcomes over a 12-month follow-up period.

3.2. Secondary Objectives

The primary secondary objective is to assess the superiority of conduction system pacing over biventricular pacing with respect to total direct medical cost. Additional secondary objectives, evaluated over a 12-month follow-up period, aim to compare both treatment arms regarding: time to first event (composite of all-cause mortality, heart failure hospitalization, or urgent heart failure visits); change in left ventricular ejection fraction (LVEF); change in left ventricular end-diastolic volume; change in Kansas City Cardiomyopathy Questionnaire (KCCQ) Overall Summary Score (OSS); change in New York Heart Association (NYHA) functional class; change in six-minute walk test (6MWT) distance; change in B-type Natriuretic Peptide (BNP) and N-terminal pro–B-type Natriuretic Peptide (NT-proBNP) levels; change in EuroQol-5D (EQ-5D) score; change in QRS duration; and a hierarchical composite endpoint including death, heart failure hospitalization, urgent HF visits, and change in KCCQ Clinical Summary Score (CSS).

4. Methods

4.1. Design

The PhysioSync-HF study is a randomized, controlled, multicenter, non-inferiority clinical trial, patient-blinded, with independent blinded adjudication of outcomes.

4.2. Randomization

Patient allocation will follow a 1:1 ratio using a randomization list generated by a blockrand function, which generates random assignments. The probability of allocation to either CRT group will be equal, and stratification will be performed by center using variable block sizes of 4 and 6 patients. The randomization list will be uploaded into the REDCap system, allowing participating sites to access the assigned treatment group only once all eligibility requirements are fulfilled. Randomization will be conducted independently by the Research Support Center of the coordinating site at Hospital Moinhos de Vento, ensuring confidentiality throughout the process.

4.3. Eligibility

4.3.1. Inclusion Criteria

- Men and women aged ≥18 years
- Symptomatic congestive heart failure (NYHA class II–III), of ischemic or non-ischemic etiology
- Left ventricular ejection fraction (LVEF) ≤35% documented by imaging within the past 3 months (echocardiogram, cardiac MRI, nuclear imaging, or left ventriculography)
- Presence of left bundle branch block (LBBB) on electrocardiogram with QRS duration ≥130 ms
- Clinical indication for cardiac resynchronization therapy (CRT), as determined by the treating physician
- Clinically stable, as assessed by the treating physician
- Receiving maximally tolerated doses of angiotensin-converting enzyme inhibitors (ACEi), angiotensin receptor blockers (ARB), or angiotensin receptor–neprilysin inhibitors (ARNI); beta-blockers; and mineralocorticoid receptor antagonists

4.3.2. Exclusion Criteria

- Heart failure classified as NYHA Class IV
- Life expectancy less than 12 months due to any condition
- Dementia or advanced cerebrovascular disease
- Planned to receive an implantable cardioverter-defibrillator (ICD) alone or with CRT
- Concurrent participation in another clinical trial involving cardiac pacing
- Pregnant women or women of childbearing potential (pre-menopausal, not using contraception)
- Inability to understand or sign the informed consent form

4.4. Outcome Definitions and Measurements

4.4.1. Primary Outcome

The primary outcome, defined as "*heart failure–related net clinical outcome,*" will be assessed as an ordinal hierarchical composite outcome compared between groups using a proportional odds model. The ordinal scale will consist of the following (in hierarchical order):

1. All-cause mortality
2. Heart failure hospitalization
3. Urgent visit due to heart failure
4. Absolute change in left ventricular ejection fraction (LVEF) from baseline to 12-month follow-up, categorized into 5% intervals (e.g., (–10, –5], (–5, 0], (0, +5], (+5, +10]).

All-cause mortality was assigned the top-weighted category in the ordinal scale, and the greatest improvement in LVEF was assigned the lowest-weighted category. The study will assess whether conduction system pacing (CSP) is non-inferior to biventricular pacing (BiVP).

The non-inferiority margin will be defined as an upper bound of the 95% confidence interval (CI) for an odds ratio <1.2. If CSP reaches the non-inferiority bound, superiority will then be tested using the same proportional odds model with alpha preservation.

4.4.2. Secondary Outcomes

The main secondary outcome is the total direct medical cost associated with conduction system pacing compared with biventricular pacing. This comparison will be conducted in the modified intention-to-treat (mITT) population, and the time frame for the analysis will correspond to the duration of clinical trial follow-up. The additional secondary outcomes, assessed up to 12 months, include:

1. Time to death, heart failure hospitalization, or urgent visit for heart failure
2. Left ventricular ejection fraction (LVEF)
3. Left ventricular end-diastolic volume
4. Kansas City Cardiomyopathy Questionnaire – Overall Summary Score (KCCQ-OSS)
5. New York Heart Association (NYHA) functional class
6. 6-minute walk test (6MWT)
7. BNP or NT-proBNP levels
8. EuroQol-5D (EQ-5D)
9. QRS complex duration
10. Hierarchical composite outcome including death, heart failure hospitalization, urgent HF visit, and change in KCCQ Clinical Summary Score (CSS)

4.4.3. Exploratory Outcomes

Exploratory outcomes, assessed up to 12 months, include:

1. Left ventricular end-systolic volume
2. Proportion of patients with improvement in KCCQ-OSS by 5, 10, and 20 points
3. Index procedure duration (minutes)
4. Index procedure complication rate
5. Implant-related adverse events
6. Cost-effectiveness
7. Cost predictors
8. Budget impact
9. Outcomes assessed by cardiopulmonary exercise testing (CPET):

    a. Peak VO_2 (primary CPET outcome)
    b. Percent predicted peak VO_2
    c. VE/VCO_2 slope
    d. Oxygen Uptake Efficiency Slope (OUES)
    e. Resting end-tidal CO_2 pressure ($PETCO_2$)
    f. Heart rate recovery at 1 minute
    g. T½ (time to 50% recovery of VO_2)

4.4.4. Safety Outcomes

Safety outcomes will be monitored throughout the entire study period, from initiation to completion. All adverse events (AEs) will be reported by the site investigators and categorized into three groups: AEs related to the index procedure, implant-related AEs, and o unrelated AEs.

4.4.5. Adjudicated Outcomes

The following expected outcomes will be reported and submitted for central adjudication: all deaths, to determine the cause of death; and all hospitalization and urgent visit events, to determine whether they were caused by heart failure. Events will be adjudicated based on the criteria detailed in the event adjudication protocol. Additionally, all echocardiograms (baseline, 6-month, and 12-month) will be submitted to an independent and blinded cardiovascular imaging core laboratory for assessment.

4.5. Sample Size

Assuming an incidence of all-cause mortality of 6.2% in both groups (based on the RAFT trial, which reported 20.8% over 40 months via linear extrapolation), a 1-year heart failure hospitalization rate of 5.85% (RAFT: 19.5% over 40 months), and a 1-year urgent visit rate for heart failure of 0.5% (DAPA-HF: 0.5 events per 100 patient-years), along with an estimated absolute increase in left ventricular ejection fraction (LVEF) of 16% in the intervention group versus 14% in the control group (a conservative estimate based on the His-Alternative study, which showed increases of 16% and 13%, respectively), and a standard deviation of 6.5 (based on values of 6 and 7 in each group from the same study), a sample size of 304 patients would be required to achieve 80% statistical power with a two-sided alpha of 5% (one-sided alpha of 2.5%) and a non-inferiority odds ratio bound <1.2 for the upper limit of the 95% confidence interval, accounting for a 5% loss to follow-up (assumed to be non-informative dropouts for the purposes of sample size calculation).

Due to slower-than-anticipated enrollment and the publication of a meta-analysis during the conduct of the PhysioSync-HF trial (Gin et al., Heart Rhythm, 2023) showing a greater improvement in LVEF in the intervention group (2.70–5.18%) than the originally assumed 2%, the Steering Committee decided to recalculate the original sample size.

The updated calculation assumed a 3% difference in LVEF improvement between groups, favoring the intervention arm. Although the originally anticipated difference based on the His-Alternative study was also 3%, a more conservative estimate of 2% had been used in the initial sample size calculation due to limited available data at the time. With the revised assumptions, the required sample size was reduced from 304 to 180 patients, while preserving the non-inferiority margin of OR less than 1.2 for the upper bound of the confidence interval and 80% statistical power. All simulations were performed using 10,000 repetitions, as detailed in Appendix A.

4.6. Interim Statistical Analyses

Early termination based on efficacy is not planned for this study. The Data and Safety Monitoring Board (DSMB) will conduct safety analyses after approximately 25%, 50%, and 75% of the target sample size has been enrolled, and at least every six months thereafter. There is no

predefined criteria for early termination. Given that the trial does not incorporate interim analyses for efficacy or safety monitoring explicitly tied to the primary endpoint, no adjustment to the alpha level will be made for the final analysis of the primary endpoint.

4.7. Timing of Final Analysis

Final analyses for all endpoints will be conducted collectively at the end of the study, after a minimum 12-month follow-up for all randomized patients and after the data lock point.

4.8. Timing of Outcome Assessments

Following CRT implantation, all patients will be evaluated in person at a follow-up visit scheduled for 30 days ± 5 days post-index procedure to assess device parameters and identify potential procedure-related complications. The primary purpose of this visit is to ensure that the patient is in a stable clinical condition and to ensure that no lead dislodgement has occurred.

Subsequent follow-up will take place at 6 months ± 20 days through a second in-person visit at the enrolling center. During this visit, a comprehensive clinical evaluation will be conducted, including a reassessment of all baseline measures: laboratory tests (including BNP or NT-proBNP), electrocardiogram, echocardiogram, Kansas City Cardiomyopathy Questionnaire (KCCQ), EQ-5D, 6-minute walk test (6MWT), and cardiopulmonary exercise testing (for those enrolled in the sub-study). The follow-up period will be calculated from the date of the index procedure. For example, follow-up day 30 is defined as 30 calendar days after the index cardiac resynchronization therapy procedure (either conduction system or biventricular pacing).

The final visit will occur at 12 months ± 20 days, during which all previously described assessments will be repeated.

5. Analysis Populations

5.1. Intention-to-Treat (ITT)

The intention-to-treat (ITT) population will include all randomized subjects, regardless of the treatment received or any violations of eligibility criteria.

5.2. Modified Intention-to-Treat (mITT)

The modified intention-to-treat (mITT) population will comprise all subjects in the ITT analysis who underwent the index procedure.

5.3. Per-Protocol (PP)

The per-protocol (PP) population will include all randomized subjects who fully adhered to the study protocol, received the treatment to which they were assigned, had no major protocol

violations (e.g., crossover to the opposite treatment arm during the study), and met all eligibility criteria at the time of randomization.

5.4. Safety Population (As-Treated)

The safety population will include all randomized subjects, analyzed according to the treatment actually received. That is, if a patient deviates from the protocol and receives the treatment opposite to their allocated arm, they will be analyzed within the group corresponding to the treatment received, regardless of initial randomization or any protocol deviations. For participants who crossed over, adverse events will be assigned to the treatment group corresponding to the therapy being received at the time of the event.

6. Principles of Statistical Analysis

The primary analysis will be conducted in the modified intention-to-treat (mITT) population. Safety outcomes will be primarily reported for the safety population, while the per-protocol (PP) population will be used for sensitivity analyses.

6.1. Confidence Intervals and p-values

The statistical significance level will be defined as a one-sided alpha of 2.5% for non-inferiority testing. For clarity in interpretation, results will be presented with two-sided 95% confidence intervals.

7. Statistical Analysis

7.1. Screening

A summary will be provided overall and by participating site, including the following variables: duration of recruitment (in days), number of patients screened, number of patients enrolled, enrollment rate (patients per day), number of screened but not enrolled patients, and reasons for non-enrollment.

7.2. Follow-Up

Patients will be followed for 12 months, with in-person visits scheduled at screening/enrollment, at the time of the index procedure, and during follow-up at 1, 6, and 12 months. The expected course of patient participation throughout the study is illustrated in the CONSORT flow diagram (**Figure 1**).

Figure 1. CONSORT flow diagram

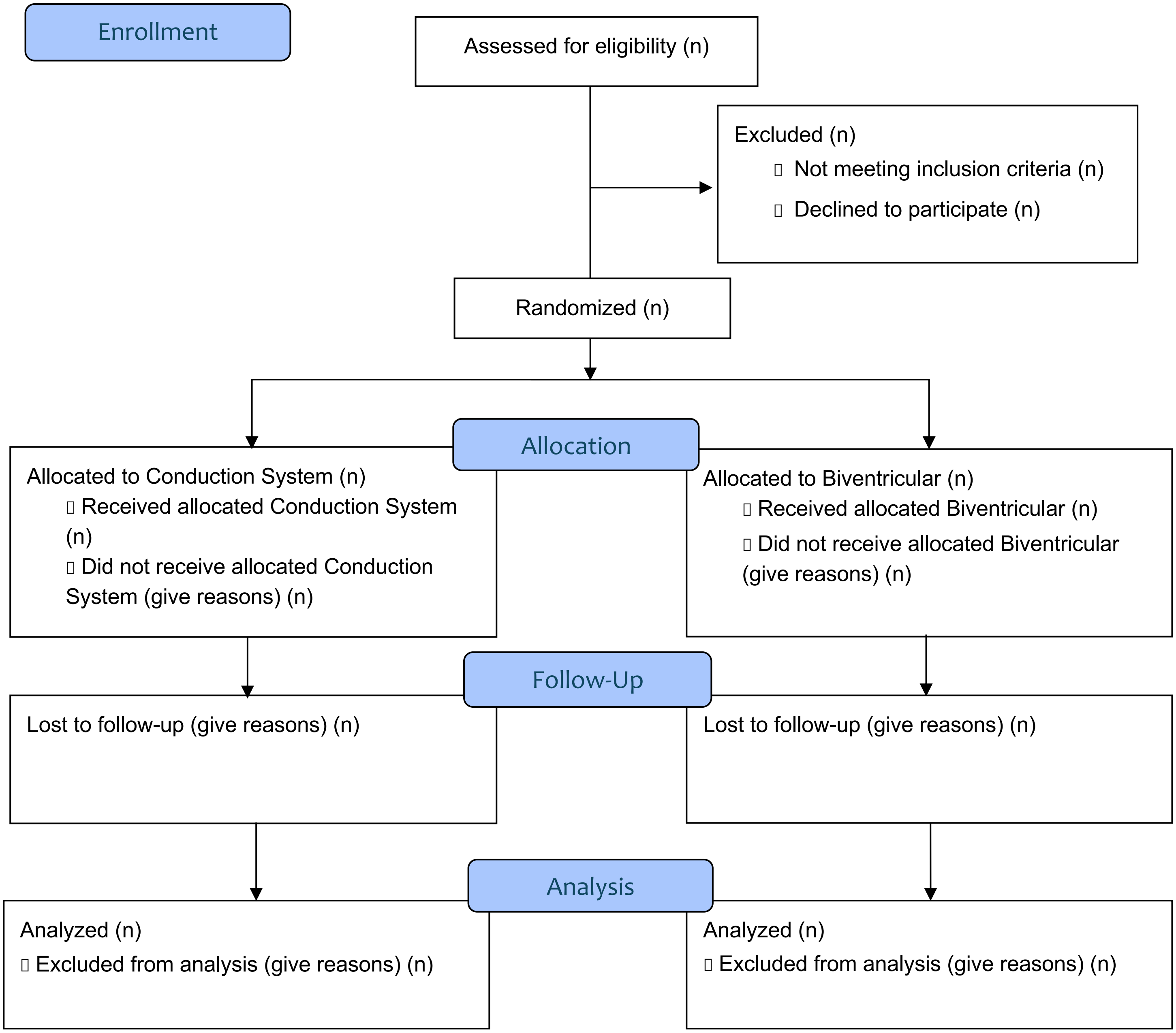

7.3. Baseline Characteristics

Information on participants' baseline characteristics will include age, sex, NYHA functional class, heart failure etiology, left ventricular ejection fraction, blood pressure, heart rate, BNP, NT-proBNP, QRS duration, and the percentage of patients using each class of medication. These data will be presented for the overall study population as well as separately for each treatment group. No statistical comparisons will be performed to avoid unnecessary hypothesis testing.

Demographic and clinical characteristics will be summarized by treatment group and for the total sample. Categorical variables will be reported as absolute numbers (n) and percentages (%), whereas continuous variables will be presented as mean and standard deviation (SD) or as median and interquartile ranges (IQR), based on the normality of data distribution.

7.4. Primary Outcome Analysis

The primary outcome, an ordinal clinical endpoint related to heart failure, will be compared between groups using a mixed-effects proportional odds model. The model will include a random effect for recruiting site and will be adjusted for baseline left ventricular ejection fraction, sex, and age. The primary comparison will be based on the conditional effect of the intervention, interpreted as the effect averaged over the distribution of covariates included in the model. Participants who are lost to follow-up or have only vital status data available at 12 months, with no clinical outcome information after randomization, will be excluded from the primary outcome analysis. If a patient has no recorded clinical events (death, hospitalization, or urgent heart failure visit) but lacks an LVEF assessment within the specified time window, another LVEF measurement collected during follow-up will be used for imputation. If no LVEF data are available, the mean 12-month change in LVEF, pooled across both treatment groups, will be imputed.

The validity of the proportional odds assumption will be tested to support the interpretation of results. However, even if the assumption is not met, the primary outcome will still be analyzed using the proposed model, and the estimated odds ratio can be interpreted as a summary (average) effect across the ordinal scale (21). Analyses will be performed using the rms package in R (22).

The non-inferiority margin for conduction system pacing compared with biventricular pacing will be defined as an odds ratio <1.20 for the upper bound of the confidence interval. If non-inferiority is demonstrated at the one-sided 2.5% significance level, superiority testing will be subsequently performed using the same model, preserving the overall alpha level.

7.5. Secondary Outcome Analysis

7.5.1. Economic Outcomes and Cost Analysis

To estimate the key secondary endpoint, a cost-minimization analysis will be conducted comparing the mean total direct medical cost per patient in the conduction system pacing (CSP) group versus the biventricular pacing (BiVP) group. Cost data will be consolidated in a patient-level database. To address missing data, a hierarchical imputation strategy will be employed, applying different methods based on the relevance of each variable and the presumed cause of the data being missing.

Key cost-driving variables will include device costs, time variables of the hospital resource use (serving as a primary *proxy* for overall resource consumption and labor resource use during the index hospitalization for the procedure), and costs associated with outcomes of interest.

A tolerance of up to 10% missing data will be allowed for time variables related to hospital resource use. If this threshold is exceeded, multiple imputation using *predictive mean matching* will be applied, based on covariates: enrollment site, baseline LVEF, sex, age, treatment group, and crossover occurrence. We will assume that missing data for these variables occur randomly at the patient level. No missing data are expected for device costs or outcome-related costs. For other cost-related variables with >10% missing data, median imputation will be used, assuming low impact on total costs and, consequently, on mean costs per treatment arm.

The primary economic endpoint will be presented as the mean cost difference between treatment groups. A generalized linear model with a log-link and gamma distribution will be used to test for significance, alongside non-parametric bootstrapping with 1,000 resamples to derive 95% confidence intervals. This approach accounts for the expected right-skewed distribution of cost data. Model adjustments may be made based on the final distribution of cost data at the end of follow-up. Additionally, median and interquartile ranges for cost outcomes will be reported by treatment group. This analysis will be based on the modified intention-to-treat (mITT) population, and the time frame will correspond to the duration of follow-up in the study.

7.5.2. Time to Death, Heart Failure Hospitalization, or Urgent HF Visit

The composite outcome comprising the primary endpoint components (all-cause death, heart failure hospitalization, and urgent heart failure visits) will be analyzed as time to first event using Cox proportional hazards models. Results will be reported as hazard ratios (HRs) with corresponding 95% confidence intervals. Kaplan-Meier estimates will be used to display unadjusted time-to-event curves. All models will incorporate a random effect for participating site (frailty model) and will be adjusted for baseline left ventricular ejection fraction, sex, and age.

7.6. Hierarchical Composite Outcome

Death, Heart Failure Hospitalization, Urgent HF Visit, and Change in KCCQ-CSS

The hierarchical ordinal composite outcome comprising death, heart failure hospitalization, urgent heart failure visit, and change in KCCQ Clinical Summary Score (KCCQ-CSS) will be analyzed using the same proportional odds logistic regression model applied for the primary outcome. In cases of missing data, imputation for KCCQ-CSS will follow the same approach used for LVEF in the primary endpoint analysis. Non-inferiority testing will not be performed for this secondary outcome.

Table 1. Outcomes Assessed at 12 Months by Treatment Allocation

Outcomes	Biventricular Pacing [control]	Conduction System Pacing [intervention]	Effect Measure	Unadjusted effect [95% CI]	Adjusted effect [95% CI]	p-value
**Primary outcome: heart failure–related clinical improvement**			Odds ratio	x.xx [x.xx - x.xx]	x.xx [x.xx - x.xx]	x.xxx
All-cause death	xx (xx%)	xx (xx%)				
HF Hospitalization	xx (xx%)	xx (xx%)				
Urgent Visit for HF	xx (xx%)	xx (xx%)				
Absolute change in LVEF at 12 months						
< -10%	xx (xx%)	xx (xx%)				
[-10% a -5%)	xx (xx%)	xx (xx%)				
[-5% a 0%)	xx (xx%)	xx (xx%)				
[0% a 5%)	xx (xx%)	xx (xx%)				
[5% a 10%)	xx (xx%)	xx (xx%)				
[10% a 15%)	xx (xx%)	xx (xx%)				
[15% a 20%)	xx (xx%)	xx (xx%)				
[20% a 25%)	xx (xx%)	xx (xx%)				
[25% a 30%)	xx (xx%)	xx (xx%)				
[30% a 35%)	xx (xx%)	xx (xx%)				
≥ 35%	xx (xx%)	xx (xx%)				
**Secondary outcomes**						
Mean direct medical cost per patient	xx (x.xx - x.xx)	xx (x.xx - x.xx)	Mean difference	x.xx [x.xx - x.xx]	-	
Composite of all-cause mortality, HF hospitalization, or urgent visit for HF	xx (xx%)	xx (xx%)	Hazard Ratio	x.xx [x.xx - x.xx]	x.xx [x.xx - x.xx]	x.xxx
All-cause mortality	xx (xx%)	xx (xx%)	Hazard Ratio	x.xx [x.xx - x.xx]	x.xx [x.xx - x.xx]	x.xxx
HF hospitalization	xx (xx%)	xx (xx%)	Hazard Ratio	x.xx [x.xx - x.xx]	x.xx [x.xx - x.xx]	x.xxx
Urgent visit for HF	xx (xx%)	xx (xx%)	Hazard Ratio	x.xx [x.xx - x.xx]	x.xx [x.xx - x.xx]	x.xxx
Secondary ordinal outcome: all-cause mortality, HF hospitalization, urgent HF visit, and change in KCCQ-CSS	xx (xx)	xx (xx)	Odds ratio	x.xx [x.xx - x.xx]	x.xx [x.xx - x.xx]	x.xxx

7.7. Natriuretic Peptides

The outcome of the relative change in natriuretic peptide levels at up to 12 months will be analyzed separately for BNP and NT-proBNP. A linear regression model will be used to assess the relative change (i.e., follow-up value divided by baseline value), with adjustments for baseline values. Results will be presented as mean differences in relative change between treatment groups, along with corresponding 95% confidence intervals. If the 12-month assessment is not available, an alternative measurement obtained during the follow-up period will be used. Patients without any measurement of the respective peptide during follow-up will be excluded from this analysis.

7.8. Functional Class

NYHA functional class at 12 months will be described using absolute and relative frequencies and compared between treatment groups using ordinal regression models similar to the one used for the primary outcome, adjusted for baseline NYHA class. Results will be reported as proportional odds ratios, consistent with the primary outcome analysis.

7.9. Other Secondary Outcomes

Secondary outcomes assessed up to 12 months will be analyzed using linear regression models adjusted for baseline values. Treatment effects will be reported as mean differences with corresponding 95% confidence intervals. If a patient does not have a 12-month outcome assessment, an alternative measurement obtained during the study follow-up will be used. Patients with no measurements for a given variable during follow-up will be excluded from that specific analysis. **Table 2** provides a proposed summary of the secondary outcome effects.

Table 2. Comparative Analysis of Secondary Outcomes by Treatment Group

Secondary Outcomes	Biventricular Pacing [control]	Conduction System Pacing [intervention]	Effect Measure	Effect [95% CI]	p-value
**Echocardiographic Outcomes**					
Ejection fraction (%)	xx.x (xx.x)	xx.x (xx.x)	Mean dif.	x.xx [x.xx - x.xx]	x.xxx
End-diastolic volume (ml)	xx.x (xx.x)	xx.x (xx.x)	Mean dif.	x.xx [x.xx - x.xx]	x.xxx
**Electrocardiographic and laboratory outcomes**					
Change in QRS duration (ms)	xx.x (xx.x)	xx.x (xx.x)	Mean dif.	x.xx [x.xx - x.xx]	x.xxx
BNP (pg/ml)	xx.x (xx.x)	xx.x (xx.x)	Mean dif.	x.xx [x.xx - x.xx]	x.xxx
NT-proBNP (pg/ml)	xx.x (xx.x)	xx.x (xx.x)	Mean dif.	x.xx [x.xx - x.xx]	x.xxx
**Quality of life and functional capacity**					
6MWT distance (m)	xx.x (xx.x)	xx.x (xx.x)	Mean dif.	x.xx [x.xx - x.xx]	x.xxx
NIHA class					
I	xx.x (xx.x)	xx.x (xx.x)	Mean dif.	x.xx [x.xx - x.xx]	x.xxx
II	xx.x (xx.x)	xx.x (xx.x)	Mean dif.	x.xx [x.xx - x.xx]	x.xxx
III	xx.x (xx.x)	xx.x (xx.x)	Mean dif.	x.xx [x.xx - x.xx]	x.xxx
IV	xx.x (xx.x)	xx.x (xx.x)	Mean dif.	x.xx [x.xx - x.xx]	x.xxx
KCCQ-OSS	xx.x (xx.x)	xx.x (xx.x)	Mean dif.	x.xx [x.xx - x.xx]	x.xxx
EQ5D	xx.x (xx.x)	xx.x (xx.x)	Mean dif.	x.xx [x.xx - x.xx]	x.xxx

7.10. Sensitivity Analyses

The primary endpoint includes, as part of the main analysis, imputation of LVEF values for patients without echocardiographic assessments during follow-up. As a first sensitivity analysis, the ordinal logistic model will be re-estimated including only patients with complete data.

As a second sensitivity analysis, the model will be refitted using the raw change in LVEF without categorization into predefined bands—that is, modeling discrete unit changes (e.g., -10%, -11%, -12%, -13%, etc.).

Additionally, to assess the robustness related to hospitalization and urgent visits for heart failure events, the total number of events during follow-up will be analyzed as count data using Poisson regression with a log-link function. These models will be adjusted for total follow-up time and the same covariates used in the Cox model. Results will be presented as rate ratios with 95% confidence intervals.

Finally, all primary and secondary efficacy analyses will also be conducted using the per-protocol (PP) and as-treated populations.

7.11. Subgroup Analyses

The primary ordinal outcome will be assessed within predefined subgroups in the mITT population by including interaction terms between treatment assignment and each subgroup variable in the same mixed-effects ordinal logistic regression model used for the primary analysis.

Treatment effects will be reported as conditional proportional odds ratios within each subgroup level, along with corresponding 95% confidence intervals. No adjustment for multiple comparisons will be applied. The following subgroups will be evaluated:

1. Age: ≥ vs. < median
2. Sex: male vs. female
3. Operator experience: ≥ vs. < 40 procedures performed before trial initiation
4. Baseline QRS duration: ≥ vs. < median
5. Baseline LVEF: ≥ vs. < median
6. Indexed end-diastolic volume: ≥ vs. < median
7. Heart failure etiology: ischemic vs. non-ischemic
8. Atrial fibrillation vs. other rhythm

7.12. Functional Capacity Sub-study

Cardiopulmonary Exercise Testing

Cardiopulmonary exercise test (CPET) outcomes up to 12 months will be analyzed using linear regression models adjusted for baseline values. Effects will be reported as mean differences between treatment groups with corresponding 95% confidence intervals.

If the CPET is not performed at the 12-month visit, an alternative assessment obtained during the follow-up period will be used. If no follow-up measurement of the variable is available, the patient will be excluded from the corresponding analysis.

7.13. Missing Data

Imputation strategies for the primary and secondary efficacy endpoints are defined within their respective analysis sections. For other exploratory outcomes, minimal missing data are anticipated given the study's structured procedures, which include site staff training and independent data monitoring.

Nevertheless, the coordinating center will contact the site investigators to obtain any missing data variables. Analyses for primary and secondary outcomes will be performed based on participants with available outcome data (i.e., available case analysis), except for the specific imputation approaches already outlined for the primary endpoint, the economic secondary endpoint, and the ordinal secondary endpoint as detailed in the Outcomes Analysis section.

7.14. Additional Definitions

For the assessment of clinical outcomes, any hospitalization during which the patient undergoes heart transplantation will be classified as a heart failure hospitalization. For endpoints not involving clinical outcomes (i.e., death, hospitalization, or urgent visit for heart failure) or costs, patients who undergo heart transplantation will be censored on the date of transplantation.

Clinical outcomes (i.e., death, hospitalization, or urgent visit for heart failure) occurring after the last day of the final scheduled follow-up period (i.e., one year and 20 days after the index procedure) will not be counted. However, all other outcomes will be considered even if the 12-month visit takes place beyond the protocol-defined window.

7.15. Statistical Software

All analyses will be conducted using R software version 4.4.1 (or later) (R Development Core Team, Vienna, Austria, www.r-project.org).

8. References

1. Kandala J, Upadhyay GA, Altman RK, Parks KA, Orencole M, Mela T, et al. QRS morphology, left ventricular lead location, and clinical outcome in patients receiving cardiac resynchronization therapy. Eur Heart J. agosto de 2013;34(29):2252–62.
2. McAlister FA, Ezekowitz J, Hooton N, Vandermeer B, Spooner C, Dryden DM, et al. Cardiac resynchronization therapy for patients with left ventricular systolic dysfunction: a systematic review. JAMA. 13 de junho de 2007;297(22):2502–14.
3. Cleland JGF, Daubert J-C, Erdmann E, Freemantle N, Gras D, Kappenberger L, et al. The effect of cardiac resynchronization on morbidity and mortality in heart failure. N Engl J Med. 14 de abril de 2005;352(15):1539–49.
4. Moss AJ, Hall WJ, Cannom DS, Klein H, Brown MW, Daubert JP, et al. Cardiac-Resynchronization Therapy for the Prevention of Heart-Failure Events. N Engl J Med. 1o de outubro de 2009;361(14):1329–38.
5. Chinitz JS, d'Avila A, Goldman M, Reddy V, Dukkipati S. Cardiac resynchronization therapy: who benefits? Ann Glob Health. fevereiro de 2014;80(1):61–8.
6. Rohde LE, Bertoldi EG, Goldraich L, Polanczyk CA. Cost-effectiveness of heart failure therapies. Nat Rev Cardiol. junho de 2013;10(6):338–54.
7. Gazzoni GF, Fraga MB, Ferrari ADL, Soliz P da C, Borges AP, Bartholomay E, et al. Preditores de Mortalidade Total e de Resposta Ecocardiográfica à Terapia de Ressincronização Cardíaca: Um Estudo de Coorte. Arq Bras Cardiol. dezembro de 2017;109(6):569–78.
8. Daubert C, Behar N, Martins RP, Mabo P, Leclercq C. Avoiding non-responders to cardiac resynchronization therapy: a practical guide. Eur Heart J. 14 de maio de 2017;38(19):1463–72.
9. Ajijola OA, Upadhyay GA, Macias C, Shivkumar K, Tung R. Permanent His-bundle pacing for cardiac resynchronization therapy: Initial feasibility study in lieu of left ventricular lead. Heart Rhythm. 2017;14(9):1353–61.
10. Huang W, Su L, Wu S, Xu L, Xiao F, Zhou X, et al. Long-term outcomes of His bundle pacing in patients with heart failure with left bundle branch block. Heart Br Card Soc. 2019;105(2):137–43.
11. Huang W, Su L, Wu S, Xu L, Xiao F, Zhou X, et al. A Novel Pacing Strategy With Low and Stable Output: Pacing the Left Bundle Branch Immediately Beyond the Conduction Block. Can J Cardiol. 1o de dezembro de 2017;33(12):1736.e1-1736.e3.
12. Sharma PS, Dandamudi G, Herweg B, Wilson D, Singh R, Naperkowski A, et al. Permanent His-bundle pacing as an alternative to biventricular pacing for cardiac resynchronization therapy: A multicenter experience. Heart Rhythm. 2018;15(3):413–20.
13. Wang Songjie, Wu Shengjie, Xu Lei, Xiao Fangyi, Whinnett Zachary I., Vijayaraman Pugazhendhi, et al. Feasibility and Efficacy of His Bundle Pacing or Left Bundle Pacing Combined With Atrioventricular Node Ablation in Patients With Persistent Atrial Fibrillation and Implantable Cardioverter-Defibrillator Therapy. J Am Heart Assoc. 17 de dezembro de 2019;8(24):e014253.
14. Zanon F, Ellenbogen KA, Dandamudi G, Sharma PS, Huang W, Lustgarten DL, et al. Permanent His-bundle pacing: a systematic literature review and meta-analysis. Eur Eur Pacing Arrhythm Card Electrophysiol J Work Groups Card Pacing Arrhythm Card Cell Electrophysiol Eur Soc Cardiol. 01 de 2018;20(11):1819–26.

15. Upadhyay GA, Vijayaraman P, Nayak HM, Verma N, Dandamudi G, Sharma PS, et al. His Corrective Pacing or Biventricular Pacing for Cardiac Resynchronization in Heart Failure. J Am Coll Cardiol. 9 de julho de 2019;74(1):157–9.
16. Sharma PS, Dandamudi G, Naperkowski A, Oren JW, Storm RH, Ellenbogen KA, et al. Permanent His-bundle pacing is feasible, safe, and superior to right ventricular pacing in routine clinical practice. Heart Rhythm. 1o de fevereiro de 2015;12(2):305–12.
17. Lustgarten DL, Crespo EM, Arkhipova-Jenkins I, Lobel R, Winget J, Koehler J, et al. His-bundle pacing versus biventricular pacing in cardiac resynchronization therapy patients: A crossover design comparison. Heart Rhythm. julho de 2015;12(7):1548–57.
18. Upadhyay GA, Tung R. His Bundle Pacing for Cardiac Resynchronization. Card Electrophysiol Clin. setembro de 2018;10(3):511–7.
19. Qian Z, Zou F, Wang Y, Qiu Y, Chen X, Jiang H, et al. Permanent His bundle pacing in heart failure patients: A systematic review and meta-analysis. Pacing Clin Electrophysiol PACE. fevereiro de 2019;42(2):139–45.
20. Upadhyay GA, Vijayaraman P, Nayak HM, Verma N, Dandamudi G, Sharma PS, et al. On-treatment comparison between corrective His bundle pacing and biventricular pacing for cardiac resynchronization: A secondary analysis of the His-SYNC Pilot Trial. Heart Rhythm. dezembro de 2019;16(12):1797–807.
21. Harrel, Jr FE (2022) Assessing the Proportional Odds Assumption and Its Impact https://www.fharrell.com/post/impactpo/
22. Harrell Jr FE (2023). rms: Regression Modeling Strategies. https://hbiostat.org/R/rms/, https://github.com/harrelfe/rms.

APPENDIX A

Four power simulations were conducted using 10,000 iterations each, assuming a total sample size of 180 patients (90 per group) and odds ratio non-inferiority margins of 1.0, 1.1, 1.2, and 1.3.

Parameters

- Alpha (two-sided) 0.05
- Primary ordinal outcome: all-cause mortality at 12 months, heart failure hospitalization at 12 months, urgent heart failure visits at 12 months, and change in left ventricular ejection fraction (LVEF) at 12 months from baseline (categorized in 5% increments)
- Expected event rates (assuming independence)
- Mortality 6.24% in both groups
- Heart failure hospitalization 5.85% in both groups
- Urgent heart failure visits 0.5% in both groups
- Mean LVEF improvement 16% in the CSP group vs 13% in the BVP group
- Standard deviation of LVEF improvement 6.5
- 5% loss to follow-up

Estimated power under different non-inferiority margins

Non-inferiority margin OR < 1.0: 57.8%
Non-inferiority margin OR < 1.1: 71.3%
Non-inferiority margin OR < 1.2: 81.4% (this was the pre-specified non-inferiority margin for the primary outcome)
Non-inferiority margin OR < 1.3: 88.3%

This *Statistical Analysis Plan* (SAP) was developed prior to the initiation of study analyses, with the aim of standardizing procedures related to the execution, processing, and reporting of statistical data, ensuring consistency and transparency in the conduct of the analyses.

[Andre Zimerman]

[Carisi Anne Polanczyk]

[Lucas Damiani]
