# Supplementary material for: Conduction System vs Biventricular Pacing in Heart Failure: The PhysioSync-HF Randomized Clinical Trial
Source: JAMA Cardiol. 2026 Mar 11;11(4):360–8. doi: 10.1001/jamacardio.2026.0101 (PMC12980360; doi:10.1001/jamacardio.2026.0101)
Supplement: Supplement 3. — eAppendix 1. Trial Leadership and Committees eAppendix 2. Site Investigators eMethods. Additional Details on Methods eTable 1. Procedural Characteristics and Complications eTable 2. Sensitivity Analyses for the Primary End Point eTable 3. Individual Causes of Death eTable 4. Subgroup Analysis for the Primary End Point eTable 5. Narrative Summaries of Deaths Associated With Procedural Complications eFigure 1. Change in Left Ventricular Ejection Fraction by Resynchronization Lead Location eFigure 2. Change in Left Ventricular Ejection Fraction at 12 Months by Presence of Terminal Positive Wave in Lead V1 [file jamacardiol-e260101-s003.pdf]

Supplemental Online Content

Zimerman A, dal Forno A, Rohde LE, et al. Conduction system vs biventricular pacing in heart failure: the PhysioSync-HF randomized clinical trial. *JAMA Cardiol*. Published online March 11, 2026. doi:10.1001/jamacardio.2026.0101

eAppendix 1. Trial Leadership and Committees
eAppendix 2. Site Investigators
eMethods. Additional Details on Methods
eTable 1. Procedural Characteristics and Complications
eTable 2. Sensitivity Analyses for the Primary End Point
eTable 3. Individual Causes of Death
eTable 4. Subgroup Analysis for the Primary End Point
eTable 5. Narrative Summaries of Deaths Associated With Procedural Complications
eFigure 1. Change in Left Ventricular Ejection Fraction by Resynchronization Lead Location
eFigure 2. Change in Left Ventricular Ejection Fraction at 12 Months by Presence of Terminal Positive Wave in Lead V1

This supplemental material has been provided by the authors to give readers additional information about their work.

© 2026 Zimerman A et al. *JAMA Cardiology*.

eAppendix 1. Trial Leadership and Committees (listed in alphabetical order)

Steering Committee
Andre d'Avila, MD, PhD, Hospital Moinhos de Vento, Hospital SOS Cardio, and Beth Israel Deaconess Medical Center
Alexander dal Forno, MD, Hospital SOS Cardio
André Zimerman, MD, PhD, Hospital Moinhos de Vento Caique Ternes, MD, PhD, Hospital SOS Cardio
Carisi A. Polanczyk, MD, PhD, Hospital Moinhos de Vento Fernanda D. Alves, PhD, Hospital Moinhos de Vento Leandro I. Zimerman, MD, PhD, Hospital Moinhos de Vento Luis E. Rohde, MD, PhD, Hospital Moinhos de Vento

Independent Data and Safety Monitoring Board
Eduardo B. Saad, MD, PhD
Fabiana Marcondes-Braga, MD, PhD Patrícia O. Guimarães, MD, PhD (Chair)

Clinical Events Committee Fernando L. Scolari, MD, PhD Luis E. Rohde, MD, PhD (Chair) Mariana G. Blacher, MD, MS

Echocardiography Core Laboratory
Luis E. Rohde, MD, PhD Willian R. Menegazzo, MD, MS

© 2026 Zimerman A et al. *JAMA Cardiology*.

eAppendix 2. Site Investigators (listed in descending order of the number of randomized patients)

Instituto do Coração, Hospital das Clínicas da Universidade de São Paulo: Martino Martinelli and Roberto Costa (Principal Investigators), Wagner Nascimento, Kátia Regina da Silva.

Hospital Ana Nery: Alexsandro Fagundes (Principal Investigator), Pollianna Roriz, Renata Balthazar.

Instituto Nacional do Coração: Rodrigo Barbosa Minatti (Principal Investigator), Gabriella Piumbini dos Santos, Valéria Gonçalves da Silva, Julianny Freitas Rafael, Lisa de Castro Fialho.

Hospital de Clínicas de Porto Alegre: Leandro Zimerman (Principal Investigator), Maurício Pimentel, Ana Paula Arbo Magalhães, Lucas Simonetto Faganello.

Instituto de Medicina Integral Professor Fernando Figueira: Eduardo Barreto Gadelha (Principal Investigator), Nathalia Santos Barbosa da Silva, Rodrigo Moreno Dias Carreiro.

Hospital Universitário da Universidade Federal do Piauí: Carlos Eduardo Batista de Lima (Principal Investigator), Cláudia Karina Guarino Lins, Lyon Richardson da Silva Nascimento, Lucas Teixeira Dias, Paulo Matheus de Oliveira Araújo, Rafael Cardoso Jung Batista.

Hospital Universitário Cassiano Antonio de Moraes: Marcio Augusto Silva (Principal Investigator), Fernando Luiz Torres Gomes, Patrick Ventorim Costa, Jorge Elias Neto, Marcelo Da Costa Maia, Guilherme Muller De Campos Futuro, Erick Sessa Merçon, Ricardo Ryoshim Kuniyoshi; Deborah Miranda De Vasconcelos.

Fundação Hospital do Coração Francisca Mendes: Jaime Arnez Maldonado (Principal Investigator).

Hospital SOS Cardio: Alexander Romeno Dal Forno (Principal Investigator), Bruna Miers May, Clovis Froemming Junior.

Hospital Geral Universitário de Cuiabá: Júlio César Oliveira (Principal Investigator), Ana Barbara Rezende, Lohrayne de Paula Borges, Samir Yoshio Matsumoto Bissi.

Instituto de Cardiologia de Santa Catarina: Fabrício Mallmann (Principal Investigator), Luciano Boff, Mauricio Spessatto, Fernando Fraiha, Crystian Josué Tholl, Gabriel Odozynski.

Instituto de Cardiologia e Transplante do Distrito Federal: José Mario Baggio Júnior (Principal Investigator), Jacqueline Pereira Oliveira.

Beneficiência Portuguesa: Carlos Eduardo Duarte (Principal Investigator), Silas dos Santos Galvão Filho, José Tarcisio Medeiros de Vasconcelos.

Hospital Moinhos de Vento: Leandro Zimerman (Principal Investigator), Maurício Pimentel, Ana Paula Arbo Magalhães, Lucas Simonetto Faganello.

© 2026 Zimerman A et al. *JAMA Cardiology*.

eMethods. Additional Details on Methods

Further information is available in the study protocol and in the published design paper (Zimerman A et al., *Am Heart J* 2025 Jun 3:290:38-45).

Eligibility Criteria

Inclusion criteria:

- Age ≥ 18 years
- Symptomatic congestive heart failure (NYHA class II–III), of ischemic or non-ischemic etiology
- Left ventricular ejection fraction ≤ 35% documented by imaging within the past 3 months (echocardiogram, cardiac MRI, nuclear imaging, or left ventriculography)
- Presence of left bundle branch block on electrocardiogram with a QRS duration ≥ 130 ms
- Clinical indication for cardiac resynchronization therapy, as determined by the treating physician
- Clinically stable, as assessed by the treating physician
- Receiving maximally tolerated doses of angiotensin-converting enzyme inhibitors, angiotensin receptor blockers, or angiotensin receptor–neprilysin inhibitors; beta-blockers; and mineralocorticoid receptor antagonists

Exclusion criteria:

- Heart failure classified as NYHA Class IV
- Life expectancy of less than 12 months due to any condition
- Dementia or advanced cerebrovascular disease
- Plan to receive an implantable cardioverter-defibrillator alone or with cardiac resynchronization therapy
- Concurrent participation in another clinical trial involving cardiac pacing
- Pregnant women or women of childbearing potential (pre-menopausal, not using contraception)
- Inability to understand or sign the informed consent form

Sample size

The sample size calculation has been described in detail previously. An initial sample size of 304 patients was calculated to provide 80% power to demonstrate the non-inferiority of conduction system pacing compared with biventricular pacing for the heart failure–related hierarchical composite outcome at 12 months. This estimate assumed, for both groups, a 5% annual loss to follow-up, an all-cause mortality rate of 6.24%, a heart failure hospitalization rate of 5.85%, an urgent heart failure visit rate of 0.5%, and an absolute mean difference in LVEF change of +2.0 percentage points favoring conduction system pacing, with a common standard deviation of 6.5.

Midway through enrolment, the Steering Committee revisited the sample size in response to slower-than-anticipated recruitment, which risked escalating trial costs, and the publication of a meta-analysis reporting a potentially greater LVEF benefit with CSP (mean difference ranging

© 2026 Zimerman A et al. *JAMA Cardiology*.

from +2.7% to +5.2%). At that time, 87 patients had been randomized, and none had reached the 12-month follow-up. Based on this emerging evidence, the sample size was recalculated assuming a +3.0% difference in LVEF favoring CSP, resulting in a revised enrolment target of 180 patients.

© 2026 Zimerman A et al. *JAMA Cardiology*.

eTable 1. Procedural Characteristics and Complications

Characteristics	Conduction System Pacing (N=87)	Biventricular Pacing (N=86)
Procedure duration, min	120 (80, 165) (n=86)	120 (80, 162) (n=86)
Fluoroscopy duration, min	20 (10, 36) (n=83)	19 (12, 29) (n=85)
Resynchronization lead location		
Left bundle branch area	55	-
His bundle	2	-
Deep septal	16	-
Left bundle branch area + coronary sinus	7	-
Coronary sinus	5	78
None	2	1
Final QRS duration, ms	120 (103, 133) (n=80)	126 (118, 138) (n=83)
Change from baseline, ms	-60 (-80, -45) (n=80)	-55 (-71, -36) (n=83)
R-wave peak time in lead V6, ms	83 (70, 105) (n=79)	-
< 90 ms	44/79	-
< 80 ms	33/79	-
V6-V1 interpeak interval, ms	42 (25, 63) (n=79)	-
> 33 ms	49/79	-
> 44 ms	38/79	-
QLV, ms	-	121 (98, 150) (n=65)
Threshold, V	0.8 (0.5, 1.0) (n=71)	0.8 (0.6, 1.4) (n=71)
Complications during follow-up	10	7
Atrial lead dislodgement	3	2
Ventricular lead dislodgement	0	4 (3 coronary sinus; 1 septal, after crossover)
Pneumothorax	2	0
Threshold rise, lead fracture	3 (1 atrial; 1 septal; 1 coronary sinus)	0
Pericardial effusion without tamponade	0	1
Perforation and tamponade	2 (1 septal; 1 coronary sinus, after crossover)	0
Device-related infection	0	0

Variables are displayed as median (interquartile range) or count. The number of patients with available information is displayed alongside each continuous value. Patients were classified according to randomized group (primary analysis population), irrespective of crossover or procedure success. Electrical outcomes were recorded at the end of the implantation procedure; complications include events accrued over the 12-month follow-up period. Resynchronization lead location was determined by the local operator. Final location was not recorded for patients from the biventricular pacing group who underwent crossover to conduction system pacing. Three patients did not receive a study device: one experienced implantation failure despite crossover to biventricular pacing, one required early termination of the procedure due to an acute complication, and one crossed over to conduction system pacing but received a dual-chamber pacemaker after the necessary materials were found to be unavailable.

© 2026 Zimerman A et al. *JAMA Cardiology*.

eTable 2. Sensitivity Analyses for the Primary End Point

Population	Odds ratio (95% CI)
Primary analysis population	2.36 (1.37–4.06)
Per-protocol	2.46 (1.38–4.39)
Excluding deaths associated with procedural complications	2.22 (1.28–3.84)

The primary analysis population included all patients who underwent the index procedure (modified intention-to-treat). The primary endpoint was a hierarchical composite that ranked patients according to the most severe event experienced over 12 months: (1) all-cause death; (2) hospitalization for heart failure; (3) urgent visit for heart failure; and (4) change in left ventricular ejection fraction from baseline to 12 months, grouped in 5% increments (i.e., 0% to <5%, 5% to <10%, etc.). CI indicates confidence interval.

© 2026 Zimerman A et al. *JAMA Cardiology*.

eTable 3. Individual Causes of Death

Randomized group	As-treated group	General cause of death	Specific cause of death
CSP	CSP	Cardiovascular	Sudden death
CSP	CSP	Cardiovascular	Procedure complication
CSP	CSP	Presumed cardiovascular	Undetermined
CSP	CSP	Non-cardiovascular	Sepsis or other infection
CSP	CSP	Non-cardiovascular	Sepsis or other infection
CSP	CSP	Non-cardiovascular	Sepsis or other infection
CSP	None	Cardiovascular	Procedure complication
CSP	BiVP	Cardiovascular	Heart failure progression
CSP	BiVP	Cardiovascular	Sudden death
CSP	BiVP	Cardiovascular	Procedure complication
CSP	BiVP	Presumed cardiovascular	Undetermined
BiVP	BiVP	Cardiovascular	Heart failure progression
BiVP	BiVP	Cardiovascular	Sudden death
BiVP	BiVP	Presumed non-cardiovascular	Undetermined
BiVP	BiVP	Non-cardiovascular	Sepsis or other infection

BiVP indicates biventricular pacing; CSP, conduction system pacing. The adjudication of each death, including the determination of whether it was related to the procedure, was performed by the blinded Clinical Events Committee.

© 2026 Zimerman A et al. *JAMA Cardiology*.

eTable 4. Subgroup Analysis for the Primary End Point

Subgroup	N of patients	Odds ratio (95% CI)	$P_{interaction}$
**Age**			0.004
< 62 years	85	1.05 (0.49 to 2.24)	
≥ 62 years	88	5.09 (2.36 to 10.99)	
**Sex**			0.90
Male	87	2.15 (1.03 to 4.49)	
Female	86	2.30 (1.07 to 4.93)	
**Heart failure etiology**			0.013
Non-ischemic	151	2.94 (1.63 to 5.32)	
Ischemic	22	0.34 (0.07 to 1.61)	
**Atrial fibrillation***			-
Yes	4	-	
No	169	2.09 (1.22 to 3.58)	
**LVEF**			0.60
< 26.3%	84	2.47 (1.13 to 5.41)	
≥ 26.3%	89	1.86 (0.90 to 3.85)	
**LVEDV**			0.95
< 193.6 mL	84	2.09 (0.97 to 4.50)	
≥ 193.6 mL	82	2.16 (0.99 to 4.73)	
**QRS duration**			0.46
< 180 msec	64	1.76 (0.74 to 4.14)	
≥ 180 msec	109	2.66 (1.34 to 5.30)	
**Prior operator experience with CSP**			0.42
< 40 procedures	74	2.89 (1.23 to 6.79)	
≥ 40 procedures	99	1.86 (0.94 to 3.66)	

Odds ratio values above 1.0 favor the biventricular pacing group. Subgroups were determined at baseline; continuous variables were stratified by the median value. All ordinal logistic regression models were adjusted for baseline LVEF and included site as a random effect. *Odds ratio and interaction not calculated due to model convergence issues caused by a low number of patients with an atrial fibrillation rhythm at the time of baseline evaluation. CSP indicates conduction system pacing; LVEDV, left ventricular end-diastolic volume; LVEF, left ventricular ejection fraction; N, number.

© 2026 Zimerman A et al. *JAMA Cardiology*.

eTable 5. Narrative Summaries of Deaths Associated With Procedural Complications

**Patient 1.** Following a successful conduction system pacing implant, the patient was extubated without complications and transferred to the coronary care unit. Approximately five hours later, the patient developed chest pain and received morphine without undergoing an echocardiographic evaluation. Subsequently, the patient experienced pulseless electrical activity arrest, unresponsive to prolonged resuscitation.
**Patient 2.** The patient was initially randomized to conduction system pacing and was undergoing crossover to biventricular pacing. Right atrial and ventricular leads had already been placed. During attempted coronary sinus cannulation, the patient developed cardiac tamponade. Pericardial drainage and surgical repair via sternotomy were performed, but the patient remained in refractory cardiogenic shock and died in the postoperative period.
**Patient 3.** During placement of a ventricular lead in the deep septum, the patient developed hypotension and loss of capture, with suspected right ventricular perforation. Initial pericardial drainage via pleuro-pericardial window led to transient stabilization. However, the patient experienced recurrent instability requiring subxiphoid and median sternotomy drainage, with removal of large clots, but no clear perforation site was identified. An epicardial left ventricular lead was ultimately implanted. The patient developed progressive multiorgan failure, including acute kidney injury and extensive venous thrombosis, and died from refractory shock in the postoperative period.

The adjudication of each death, including the determination of whether it was related to the procedure, was performed by the blinded Clinical Events Committee.

© 2026 Zimerman A et al. *JAMA Cardiology*.

eFigure 1. Change in Left Ventricular Ejection Fraction by Resynchronization Lead Location

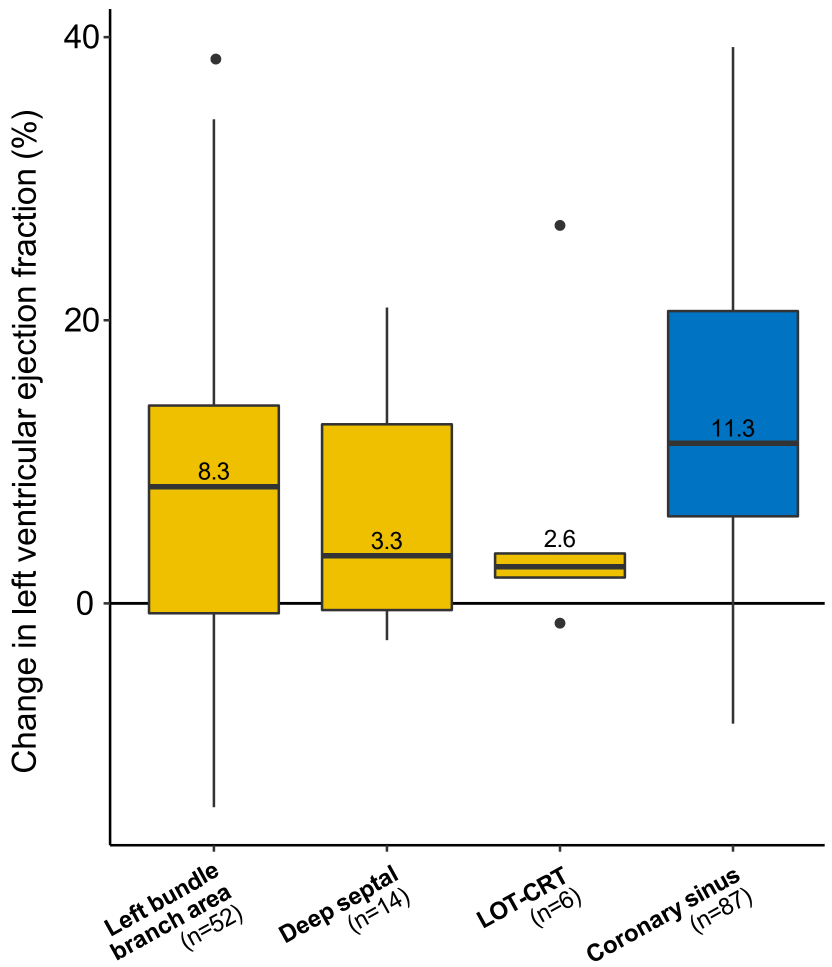

Resynchronization lead location at the end of the procedure was determined by the local operator. Blue indicates patients who were randomized to and received biventricular pacing, yellow indicates patients who were randomized to and received conduction system pacing. Box plots show the median (horizontal line), interquartile range (box), and minimum/maximum values excluding outliers (whiskers); individual outliers are plotted as points. His-bundle pacing not shown due to small number of cases (N=2). Follow-up was up to 12 months after the index procedure. LOT-CRT indicates left bundle branch area plus coronary sinus.

© 2026 Zimerman A et al. *JAMA Cardiology*.

eFigure 2. Change in Left Ventricular Ejection Fraction at 12 Months by Presence of Terminal Positive Wave in Lead V1

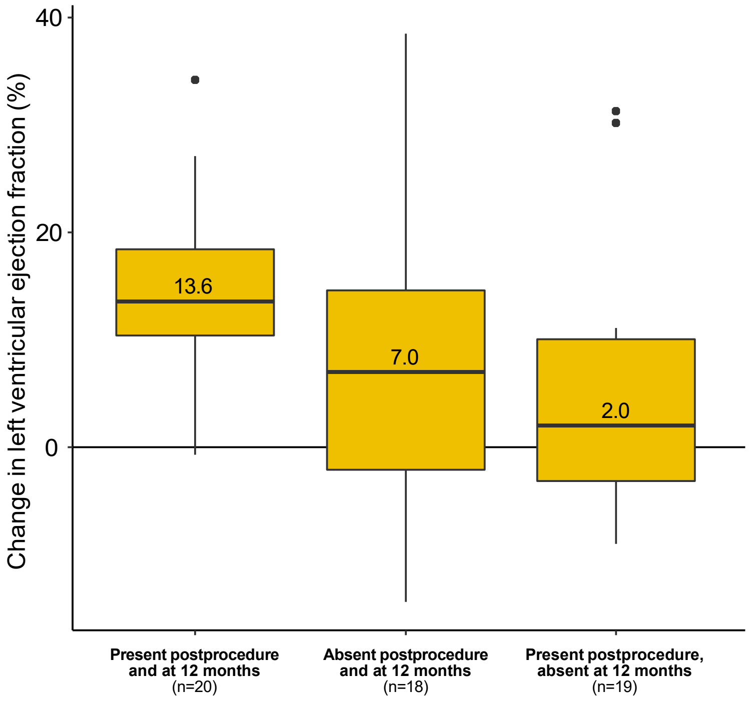

Presence of terminal positive wave in lead V1 was determined by the blinded core laboratory based on a 12-lead ECG. Analysis restricted to patients randomized to CSP who did not cross over during follow-up. Patients with a coronary sinus lead were excluded. Box plots show the median (horizontal line), interquartile range (box), and minimum/maximum values excluding outliers (whiskers); individual outliers are plotted as points. Not shown are 2 cases with terminal positive wave in V1 determined to be absent postprocedure and present at 12 months.

© 2026 Zimerman A et al. *JAMA Cardiology*.
