# Supplementary material for: Conduction System vs Biventricular Pacing in Heart Failure: The PhysioSync-HF Randomized Clinical Trial
Source: JAMA Cardiol. 2026 Mar 11;11(4):360–8. doi: 10.1001/jamacardio.2026.0101 (PMC12980360; doi:10.1001/jamacardio.2026.0101)
Supplement: Supplement 4. — Data Sharing Statement [file jamacardiol-e260101-s004.pdf]

Data Sharing Statement

Zimerman. Conduction System vs Biventricular Pacing in Heart Failure. *JAMA Cardiol*.
Published March 11, 2026. doi:10.1001/jamacardio.2026.0101

Data
Additional Information: Clinicaltrials.gov: NCT05572736
Data available: No

Additional Information
Explanation for why data not available: Because the PhysioSync-HF trial was funded by the Brazilian Ministry of Health, the data are subject to national governance policies. Interested researchers may contact the corresponding author to explore potential data access in accordance with applicable regulations.
